# Mapping bioenergetic mechanisms across aging hallmarks: a systematic evidence map and secondary conceptual synthesis

**DOI:** 10.3389/fphys.2026.1868353

**Published:** 2026-06-11

**Authors:** Torsak Tippairote, Pruettithada Hoonkaew, Aunchisa Suksawang, Prayfan Tippairote

**Affiliations:** 1 Department of Life and Consumer Science, University of South Africa, Pretoria, South Africa; 2 Thailand Initiatives for Functional Medicine, Bangkok, Thailand; 3 HP Medical Center, Bangkok, Thailand; 4 Department of Diagnostic Imaging, Mahachai Hospital, Samut Sakhorn, Thailand

**Keywords:** aging hallmarks, bioenergetics, biological resilience, evidence mapping, inflammaging, mitochondrial dysfunction, nutrient sensing, redox imbalance

## Abstract

**Background:**

Mitochondrial dysfunction is widely recognized as a feature of aging, but the term encompasses heterogeneous processes, including altered energy production, redox imbalance, substrate handling, respiratory capacity, and mitochondrial quality control. Biological resilience depends on the capacity to respond to stressors, restore homeostasis, and preserve maintenance and repair. However, within mechanism-oriented aging literature, how mitochondrial and bioenergetic mechanisms are represented across aging hallmark domains, and whether they can be organized along a continuum of impairment, remains incompletely defined.

**Methods:**

This study used a secondary evidence map and narrative synthesis based on a previously curated, mechanism-oriented literature dataset; it was not designed as a comprehensive hallmark-by-hallmark systematic review. The original search captured stress adaptation, bioenergetic regulation, systemic dysfunction, and metabolic strain. Included reports were recategorized by hallmark domain, stage of mitochondrial or bioenergetic impairment, mechanistic domain, evidence type, and evidence tier. The final synthesis included 433 reports.

**Results:**

Within this mechanism-enriched dataset, mapped evidence was concentrated in regulatory and systems-level hallmark domains, particularly altered intercellular communication, chronic inflammation, and deregulated nutrient sensing. The most frequent mechanistic labels involved redox imbalance, substrate reallocation, oxidative throughput limitation, limited adenosine triphosphate availability, and mitochondrial quality-control impairment. Stage-based mapping showed that functional, adaptive, and structural labels often coexisted across hallmark domains rather than forming discrete categories.

**Conclusion:**

These findings identify recurrent co-representation of mitochondrial and bioenergetic mechanisms with selected aging hallmark domains visible within a mechanism-oriented evidence set. The results support a cautious, hypothesis-generating interpretation in which bioenergetic constraint may provide a conceptual lens for organizing stage-like patterns, rather than reducing mitochondrial aging biology to a binary distinction between “function” and “dysfunction.” However, this analysis does not establish biological centrality, causal direction, temporal sequence, tissue specificity, or generalizability across the broader aging literature.

**Systematic Review Registration**: https://www.crd.york.ac.uk/PROSPERO/view/, identifier CRD420251033154.

## 1 Introduction

Mitochondria are important regulators of cellular and systemic homeostasis, extending beyond their canonical role in adenosine triphosphate (ATP) production to include redox regulation, metabolic integration, intracellular signaling, stress adaptation, and intercellular communication (Mootha et al., 2003; Calvo and Mootha, 2010; Cohen, 2014; Picard and Shirihai, 2022). Mitochondrial signaling is highly dynamic and context-dependent, allowing cells to adjust energy production, substrate use, calcium handling, apoptotic sensitivity, and stress-response pathways in response to changing physiological demands (Mochly-Rosen et al., 2012; Skalka et al., 2024). Mitochondrial dysfunction remains one of the most consistently observed features across aging tissues and age-related diseases (López-Otín et al., 2013; Sun et al., 2016). However, the term remains mechanistically imprecise. It is often used as an umbrella descriptor for heterogeneous alterations, including reduced ATP production, impaired respiratory reserve, redox imbalance, increased reactive oxygen species, altered substrate oxidation, and defective mitochondrial quality control (Brand and Nicholls, 2011; Nicholls and Ferguson, 2013; Wallace, 2013; Picard et al., 2016). This broad usage can obscure whether observed mitochondrial changes represent early functional constraint, compensatory adaptation, structural damage, or a mixture of these processes.

Recent work has further emphasized that mitochondria should not be interpreted through a binary framework of “function” versus “dysfunction.” Mitochondria are multifunctional, cell-type-specific, and dynamically recalibrated in response to metabolic and environmental conditions (Monzel et al., 2023). Changes in mitochondrial properties may therefore reflect adaptive shifts in molecular features, enzymatic activities, integrated functions, or organelle-level behaviors rather than irreversible damage alone. Greater conceptual precision is needed to distinguish state-dependent bioenergetic limitations from persistent structural impairment and to clarify how mitochondrial alterations relate to broader aging processes.

The aging hallmarks framework provides useful architecture for organizing these processes. The original and expanded hallmark models describe aging as a networked biological process involving molecular damage, stress-response pathways, metabolic regulation, cellular senescence, impaired communication, inflammation, dysbiosis, extracellular matrix remodeling, and broader contextual influences, including psychosocial isolation (López-Otín et al., 2013, 2023; Kroemer et al., 2025). In the present review, psychosocial isolation is acknowledged as part of this expanded geroscience context but was not included as a primary mapping domain because the analysis focused on biological hallmark domains in which mitochondrial and bioenergetic mechanisms could be directly coded. These biological hallmarks are increasingly understood as interconnected rather than independent. However, the bioenergetic basis of this interconnection remains incompletely defined.

One possible approach is to interpret mitochondrial impairment as a staged bioenergetic continuum. Early changes may involve functional constraints, such as reduced oxidative throughput, altered redox balance, impaired respiratory reserve, or limited ATP availability. These changes may trigger adaptive responses, including substrate reallocation, altered nutrient sensing, inflammatory signaling, glycolytic shift, or changes in intercellular communication. With persistent or unresolved stress, these adaptive states may become associated with structural impairment, including mitochondrial DNA damage, impaired quality control, organelle remodeling, senescence, proteostatic failure, or tissue-level decline (Picard et al., 2016; Sun et al., 2016; Spinelli and Haigis, 2018). This staged interpretation may help clarify how mitochondrial alterations are described in relation to multiple aging hallmark domains without assuming a single linear or universal pathway.

From a stress-adaptation and exposome perspective, biological systems must continuously allocate finite bioenergetic resources toward immediate survival, repair, maintenance, and long-term function (Sterling and Eyer, 1988; McEwen, 1998; McEwen and Wingfield, 2003; Rappaport, 2016). Persistent environmental, metabolic, inflammatory, or psychosocial stress may increase energetic demand while limiting the capacity for full recovery. Because mitochondrial bioenergetics supports both adaptive stress responses and restoration of homeostasis, constrained mitochondrial processing capacity may be reported alongside unresolved stress signaling, impaired repair, altered substrate handling, and progressive loss of physiological resilience (Picard et al., 2018; Picard and McEwen, 2018; Spinelli and Haigis, 2018). Recent conceptual work has proposed bioenergetic limitation as a potential organizing framework for diverse manifestations of physiological decline (Tippairote et al., 2025). In this context, the present review does not assume this framework to be correct but evaluates whether the existing literature shows recurring patterns consistent with such an interpretation.

Despite extensive investigation across mitochondrial biology and aging, it remains unclear whether mitochondrial and bioenergetic mechanisms recur across multiple aging hallmark domains, whether they can be organized by stage of impairment, and whether the literature supports an integrated or fragmented interpretation. Accordingly, this secondary evidence map reclassifies a previously curated, mechanism-oriented dataset to examine how mitochondrial and bioenergetic mechanisms are represented in relation to aging hallmark domains, with particular attention to core mechanisms, stage distribution, evidence type, and cross-domain co-representation. This framing is directly relevant to biological resilience because recovery from stress requires coordinated energy production, redox control, substrate handling, inflammatory resolution, and repair capacity across tissues. Because the analysis uses a previously curated, mechanism-oriented dataset, the review should be interpreted as a secondary evidence map of visible mitochondrial and bioenergetic co-representation, not as a comprehensive hallmark-by-hallmark systematic review.

## 2 Study objectives and research questions

### 2.1 Primary objective

To systematically map reports within a previously curated, mechanism-oriented dataset describing mitochondrial and bioenergetic mechanisms in relation to aging hallmark domains, with particular attention to whether reported mechanisms show recurring patterns consistent with a functional–adaptive–structural organizing framework.

### 2.2 Secondary objectives

- To identify, within the curated dataset, reports detailing mitochondrial or bioenergetic mechanisms relevant to aging hallmark domains, including redox imbalance, ATP limitation, oxidative throughput limitation, substrate reallocation, and mitochondrial quality control impairment.
- To classify reported mitochondrial alterations according to stage of impairment: functional, adaptive, or structural.
- To map the distribution of mitochondrial and bioenergetic mechanisms across aging hallmark domains.
- To evaluate the extent to which mechanistic labels co-occur across reports, particularly among redox imbalance, substrate reallocation, oxidative throughput limitation, ATP limitation, inflammation, intercellular communication, and deregulated nutrient sensing.
- To characterize the evidence base by report type and biological model, including review-based, human, animal, and cellular evidence.
- To identify underrepresented hallmark domains, mechanistic areas, and study designs requiring further investigation.

### 2.3 Research questions

1. What mitochondrial and bioenergetic mechanisms are most frequently described in relation to aging hallmark domains within the curated mechanism-oriented dataset?
2. How are reported mitochondrial mechanisms distributed across functional, adaptive, and structural stages of impairment?
3. Which aging hallmark domains are most frequently co-represented with mitochondrial and bioenergetic mechanisms in the included reports?
4. Which mechanistic and hallmark labels most commonly co-occur across the included reports?
5. What types of evidence — review-based, human, animal, or cellular — contribute to the curated evidence base on mitochondrial mechanisms and aging hallmarks?
6. What gaps remain in the literature, particularly regarding underrepresented hallmark domains, mitochondrial mechanisms, longitudinal evidence, and interventional studies?

## 3 Materials and methods

### 3.1 Protocol and registration

The present secondary systematic evidence map was conducted and reported in accordance with PRISMA 2020 and was registered with PROSPERO under registration number CRD420251033154. The present manuscript represents a systematic evidence map and secondary conceptual synthesis of a curated systematic dataset, which was originally developed to examine stress adaptation, bioenergetic regulation, systemic dysfunction, and biomarker patterns of metabolic strain. For the present analysis, this dataset was re-mapped to explore reports detailing mitochondrial and bioenergetic mechanisms in relation to aging hallmark domains. No additional database searches were performed beyond the original search and citation searching described below. Therefore, the present study necessarily inherits the scope and limitations of the original mechanism-oriented search. As a result, the dataset is likely enriched for reports that explicitly use mitochondrial, metabolic, bioenergetic, stress-adaptation, inflammatory, or systemic-dysfunction terminology, whereas hallmark-specific literature framed through alternative vocabularies may be underrepresented.

Any amendments to the original protocol are reported transparently as methodological adaptations. The main protocol adaptations were reclassification of the previously curated dataset according to aging hallmark domains, addition of the functional–adaptive–structural staging framework, and use of descriptive co-occurrence mapping rather than quantitative synthesis.

### 3.2 Study design

This study was designed as a structured evidence map and secondary conceptual synthesis. Its aim was to identify, classify, and synthesize reports describing mitochondrial and bioenergetic mechanisms in relation to aging hallmark domains.

The purpose was not to estimate pooled effect sizes or test a single intervention-outcome relationship. Instead, the review examined how mitochondrial mechanisms are represented within the curated mechanism-oriented dataset and whether reported mechanisms could be organized according to stage of impairment, mechanistic domain, hallmark domain, evidence type, and biological model.

This evidence-map approach was selected because the included literature was expected to be heterogeneous, including human, animal, cellular, review-based, and conceptual reports with diverse outcome measures and levels of mechanistic detail.

### 3.3 Conceptual framework

The analytical framework integrated two complementary perspectives.

First, reported mitochondrial impairment was classified using a dynamic process that distinguished functional limitation, adaptive compensation, and structural disruption. Within this framework, early functional changes may include reduced oxidative capacity, impaired respiratory reserve, ATP limitation, redox imbalance, or altered substrate oxidation. Adaptive responses may include substrate reallocation, metabolic inflexibility, altered nutrient sensing, inflammatory signaling, glycolytic shift, stress-response activation, or changes in intercellular communication. Structural changes may include mitochondrial DNA damage, impaired mitochondrial quality control, organelle remodeling, loss of proteostasis, extracellular matrix remodeling, cellular senescence, stem cell exhaustion, or tissue-level degeneration.

Second, included reports were mapped to aging hallmark domains. Domains considered during evidence mapping included genomic instability, telomere attrition, epigenetic alterations, loss of proteostasis, disabled macroautophagy, deregulated nutrient sensing, mitochondrial dysfunction as the entry domain, cellular senescence, stem cell exhaustion, altered intercellular communication, chronic inflammation, dysbiosis, and extracellular matrix remodeling.

Rather than treating mitochondrial dysfunction only as a discrete aging hallmark, this review examined whether specific mitochondrial and bioenergetic mechanisms—such as redox imbalance, ATP limitation, substrate reallocation, oxidative throughput limitation, and mitochondrial quality control impairment—were represented across multiple aging hallmark domains.

In this review, “bioenergetic constraint” is used as an operational interpretive term, not as a directly measured outcome. It refers to a reported or inferred condition in which mitochondrial or cellular energy-processing capacity appears insufficient relative to physiological demand, reflected by reported features such as impaired oxidative throughput, altered redox balance, limited ATP availability, reduced respiratory reserve, substrate rerouting, or impaired energy-dependent maintenance and repair. These features were coded, when reported, as specific mechanistic labels. The broader term “bioenergetic constraint” was used only in the Discussion and Conclusion as a hypothesis-generating synthesis of recurrent co-labeling patterns, not as an independent analytical category or causal claim.

### 3.4 Eligibility criteria

Reports were eligible for inclusion if they met the following criteria.

#### 3.4.1 Study type and scope

Eligible reports included original research studies, systematic reviews, narrative reviews, and conceptual or theoretical papers relevant to mitochondrial biology, bioenergetics, stress adaptation, aging biology, or systemic physiological decline. Original research could include observational, interventional, translational, animal, cellular, or mechanistic reports.

#### 3.4.2 Biological model

Eligible reports included human reports, animal models, and cellular or *in vitro* reports. Human studies could include clinical, population-based, interventional, or observational designs. Animal and cellular studies were included when they addressed mechanisms relevant to aging, metabolic adaptation, mitochondrial regulation, or systemic physiological stress.

#### 3.4.3 Mitochondrial or bioenergetic relevance

Reports were eligible if they described mechanisms relevant to mitochondrial function or bioenergetic regulation, including oxidative phosphorylation, respiratory capacity, redox regulation, ATP production, NAD^+^/NADH balance, substrate utilization, metabolic flux, mitochondrial quality control, mitochondrial dynamics, mitonuclear signaling, mitochondrial stress responses, oxidative stress, or energy-dependent maintenance and repair.

Reports were excluded if they mentioned mitochondrial dysfunction only generically without describing a relevant mechanism, or if they lacked a clear mitochondrial, metabolic, or bioenergetic component.

#### 3.4.4 Aging hallmark or system-level relevance

Reports were required to address systemic physiological adaptation or at least one process relevant to aging hallmarks. Eligible domains included genomic stability, epigenetic regulation, proteostasis, autophagy, nutrient sensing, cellular senescence, stem cell function, inflammation, intercellular communication, extracellular matrix remodeling, dysbiosis, metabolic adaptation, or functional decline.

Disease-specific studies were included when they provided mechanistic insight relevant to broader aging biology, systemic adaptation, or hallmark-related processes. Reports focused exclusively on disease-specific outcomes without relevance to systemic or aging-related mechanisms were excluded.

### 3.5 Information sources and search strategy

The literature dataset used in this review was derived from a systematic search designed to capture four interrelated conceptual domains:

1. stress adaptation and systemic strain;
2. bioenergetic and metabolic trade-offs;
3. system-level dysfunction across physiological domains; and
4. biomarker patterns associated with metabolic stress and functional impairment.

The original search strategy was intentionally broad and mechanism-oriented rather than restricted to aging hallmark terminology. It was designed to capture literature at the intersection of stress adaptation, bioenergetic regulation, systemic dysfunction, and biomarker patterns of metabolic strain. For the present evidence map, included reports were subsequently reclassified according to mitochondrial and bioenergetic mechanisms, stage of impairment, and aging hallmark domains.

Because the present review was not designed as a comprehensive hallmark-by-hallmark systematic review or catalogue, the search strategy directly shaped the evidence map. Reports using mitochondrial, metabolic, bioenergetic, inflammatory, senescence-related, or stress-adaptation terminology were more likely to be captured, whereas hallmark-specific studies framed primarily through telomere biology, chromatin regulation, extracellular matrix remodeling, microbiome biology, tissue-specific degeneration, or other non-bioenergetic vocabularies may have been missed.

Database searches were conducted in PubMed, Scopus, Web of Science Core Collection, and the Cochrane Library. The final database search was completed on May 15, 2025. Search strategies combined controlled vocabulary, where available, and free-text terms related to chronic stress, resilience, allostatic load, energy metabolism, mitochondrial function, substrate allocation, inflammation, senescence, sarcopenia, oxidative stress, and metabolic biomarkers. Database searches were limited to reports published between 2005 and 2025. Reports published after the final database search were not added through a new systematic database search, but a limited number were retained when identified through citation searching, final update checks, or evidence-map verification and when they provided high-yield mechanistic context directly relevant to interpretation. Although PubMed and Cochrane searches applied human-subject and language filters consistent with the original clinical/systemic review scope, animal and cellular reports were eligible when identified through Scopus, Web of Science, citation searching, final update checks, or evidence-map verification and when they provided direct mechanistic relevance to the review questions.

Forward and backward citation searching was conducted on high-yield reports to identify additional mechanistic or conceptual literature relevant to mitochondrial mechanisms, bioenergetic impairment, and aging hallmark mapping. Citation searching identified additional records that were screened and assessed using the same eligibility framework. A limited number of foundational mechanistic reports published before 2005, as well as newly available reports published after the final database search, were retained when identified through citation searching, final update checks, or evidence-map verification, provided that they offered high-yield mechanistic context directly relevant to mitochondrial bioenergetic mechanisms, aging hallmark domains, or interpretation of the functional–adaptive–structural staging framework. These reports were not used to expand the database search window systematically but were retained to preserve conceptual and mechanistic completeness within the evidence map.

The full search strategy for each database, including filters and limits, is provided in **Supplementary Table 1**.

### 3.6 Study selection and screening process

Search results were imported into Rayyan for de-duplication and screening management. Duplicate records were removed before screening. Records marked as ineligible before screening were recorded in the PRISMA flow diagram. Rayyan was used to facilitate organization, de-duplication, and screening; eligibility decisions were made by reviewers and were not replaced by automated classification.

A two-stage screening process was conducted by two independent reviewers. First, titles and abstracts were screened against predefined eligibility criteria. Second, full-text reports were retrieved and assessed for eligibility based on the inclusion and exclusion criteria and conceptual relevance to the study objectives.

Disagreements between reviewers were resolved through discussion. When consensus could not be reached, a third reviewer adjudicated. Reports that could not be retrieved after reasonable effort were classified as not retrieved and excluded from full-text assessment.

Exclusion reasons were not systematically recorded for all records excluded during title/abstract screening; therefore, the PRISMA diagram reports aggregate exclusion counts for this phase. **Supplementary Table 2** provides selected examples of excluded reports with reasons, focusing on reports that appeared potentially eligible or required closer adjudication, and should not be interpreted as a complete exclusion-reason log for all excluded records.

### 3.7 Data extraction

Data were extracted using a standardized and piloted extraction form. Extraction was performed independently by two reviewers, and discrepancies were resolved by consensus. Coding decisions were reviewed through consensus checking, and disagreements or uncertain cases were logged during extraction. Unresolved cases were adjudicated by a third reviewer. Formal inter-rater agreement statistics were not calculated because the labeling framework used non-mutually exclusive, context-dependent categories rather than mutually exclusive diagnostic classes. Most disagreements involved boundary decisions between adaptive and structural labels, especially for inflammation, nutrient sensing, mitochondrial quality control, and senescence-related processes. Representative ambiguous cases and final coding decisions are provided in **Supplementary Table 3**.

Extracted variables included:

- bibliographic information: first author, publication year, journal, and report type;
- study characteristics: design, population or model system, sample size where applicable, and setting;
- biological model: human, animal, cellular, review-based, or conceptual;
- mitochondrial and bioenergetic variables: oxidative phosphorylation, respiratory capacity, ATP production, redox status, NAD^+^/NADH balance, substrate utilization, metabolic flux, mitochondrial quality control, mitochondrial dynamics, or oxidative stress;
- aging hallmark-related processes: hallmark domain or system-level process assessed;
- mechanistic findings: pathways linking mitochondrial or bioenergetic alteration to biological outcome;
- clinical, molecular, cellular, or functional outcomes, where applicable;
- interventions, exposures, confounders, and study limitations, where reported;
- reviewer notes relevant to classification.

Missing or unclear information was recorded as “not available.” When a report included multiple relevant biological systems, hallmark domains, or mechanistic domains, all applicable labels were retained.

The data extraction template is provided in **Supplementary Table 3A**.

### 3.8 Evidence labeling framework

Following data extraction, each included report was classified using a structured, multi-dimensional evidence-labeling framework. Labels were not mutually exclusive; therefore, a single report could receive multiple stage, mechanistic, hallmark, and evidence-type labels. The labeling framework included four primary analytical dimensions: stage of mitochondrial or bioenergetic impairment, mechanistic domain, aging hallmark domain, and evidence type.

When a process could function both as a stage-related feature and as a hallmark domain, stage labels were assigned according to the role of the process in the report’s mechanistic narrative, whereas hallmark labels were assigned according to the broader aging domain represented. This distinction was used to reduce circular interpretation between stage classification and hallmark mapping.

To reduce circular interpretation, labels were assigned from the reported role of the process rather than from the biological term alone. Processes such as inflammation, nutrient sensing, intercellular communication, mitophagy, and mitochondrial quality control were therefore not automatically assigned to a fixed stage. For example, inflammatory signaling was coded as adaptive when described as a stress-responsive or compensatory response to mitochondrial or bioenergetic strain, but chronic inflammation was coded as a hallmark domain when it represented the broader aging-related domain under study. Similarly, mitophagy activation or mitochondrial quality-control remodeling was coded as adaptive when presented as compensatory organelle renewal, whereas impaired mitophagy, failed mitochondrial turnover, or accumulation of damaged mitochondria was coded as structural when presented as persistent organelle-level disruption. When a report described more than one role, multiple non-mutually exclusive labels were retained. Ambiguous coding examples and decision rules are provided in **Supplementary Table 3**.

#### 3.8.1 Stage of mitochondrial or bioenergetic impairment

Reports were categorized according to whether their findings aligned with one or more of the following stages.

Functional stage: reports describing altered mitochondrial or bioenergetic performance without clear structural damage. Examples included reduced oxidative capacity, impaired respiratory reserve, ATP limitation, redox imbalance, altered NAD^+^/NADH state, impaired substrate oxidation, or reduced metabolic flexibility.

Adaptive stage: reports describing compensatory, transitional, or stress-responsive changes in response to mitochondrial or bioenergetic strain. Examples included substrate reallocation, metabolic inflexibility, glycolytic shift, altered nutrient sensing, inflammatory signaling, stress-response activation, mitonuclear signaling, or altered intercellular communication.

Structural stage: reports describing persistent cellular, organelle-level, or tissue-level disruption. Examples included mitochondrial DNA damage, impaired mitochondrial quality control, altered mitochondrial dynamics, organelle remodeling, loss of proteostasis, extracellular matrix remodeling, cellular senescence, stem cell exhaustion, or tissue degeneration.

#### 3.8.2 Mechanistic domain

Reports were assigned one or more mechanistic labels according to the mitochondrial or bioenergetic processes described. Core mechanistic domains included:

- redox imbalance;
- substrate reallocation or metabolic inflexibility;
- oxidative throughput limitation or impaired oxidative capacity;
- ATP limitation;
- mitochondrial quality control impairment.

Additional mechanistic labels, including calcium dysregulation, mitochondrial dynamics, environmental inputs, mitonuclear signaling, and oxidative stress, were recorded where relevant. However, only the most recurrent core mechanistic domains were displayed in the main mechanistic distribution figure.

#### 3.8.3 Aging hallmark domain

Reports were mapped to one or more aging hallmark domains, including altered intercellular communication, chronic inflammation, deregulated nutrient sensing, loss of proteostasis, cellular senescence, genomic instability, stem cell exhaustion, epigenetic alterations, extracellular matrix remodeling, disabled macroautophagy, dysbiosis, and telomere attrition.

Because mitochondrial dysfunction constituted the primary mitochondrial/bioenergetic entry domain for inclusion and mechanistic classification, it was not treated as a separate final hallmark outcome in the main evidence-map figures.

#### 3.8.4 Evidence type

Reports were categorized by evidence type as review, human, animal, or cellular evidence. These categories were not mutually exclusive. For example, a review could synthesize human and animal evidence, and an original report could include more than one experimental model.

The full evidence-labeling framework and operational definitions are provided in **Supplementary Tables 3B, E**.

### 3.9 Evidence tiering

To contextualize the strength of mechanistic inference, included reports were also categorized using a four-level evidence-tier framework. This framework assessed the degree to which each report supported a relationship between mitochondrial or bioenergetic mechanisms and aging-related processes.

Tier 1 – Direct evidence: reports directly linking mitochondrial or bioenergetic mechanisms to aging-related processes through experimental, longitudinal, interventional, or clearly mechanistic evidence.
Tier 2 – Mechanistic support: reports describing mitochondrial, metabolic, redox, substrate-handling, ATP-related, or quality-control mechanisms relevant to aging or systemic adaptation, but without directly demonstrating causal or temporal progression across stages of impairment.
Tier 3 – Downstream associations: reports describing aging-related outcomes, systemic dysfunction, inflammation, frailty, senescence, functional decline, or disease-related changes with indirect or limited mechanistic linkage to mitochondrial or bioenergetic impairment.
Tier 4 – Contextual evidence: reviews, conceptual frameworks, or background literature providing theoretical or interpretive support.

Tier classification was performed independently by two reviewers, with discrepancies resolved through discussion and consensus. The evidence-tier framework was used to contextualize mechanistic contribution and inferential strength and was not designed as a formal certainty-of-evidence rating.

The evidence-tiering framework is provided in **Supplementary Table 3C**.

### 3.10 Data synthesis and evidence mapping

Because of substantial heterogeneity in report type, biological model, outcome measures, and mechanistic focus, quantitative meta-analysis was not performed. No pooled effect estimates were calculated. This heterogeneity supported the use of structured evidence mapping and narrative synthesis rather than quantitative meta-analysis.

Instead, a structured narrative synthesis and descriptive evidence map were generated. Synthesis was organized across the following dimensions:

- evidence type and biological model;
- aging hallmark domain;
- stage of mitochondrial or bioenergetic impairment;
- mechanistic domain;
- evidence tier.

The synthesis focused on identifying recurring mitochondrial mechanisms, distributional patterns across hallmark domains, stage-related patterns of impairment, label co-occurrence, and evidence gaps.

Descriptive counts and proportions were calculated for non-mutually exclusive labels. Because reports could receive multiple labels, counts represent labeled report associations rather than mutually exclusive study totals.

### 3.11 Graphical and network visualization methods

Evidence-map findings were displayed using horizontal bar charts, proportional stacked bar charts, absolute stacked bar charts, and a co-occurrence network.

Horizontal bar charts were used to summarize evidence type and core mechanistic-domain frequencies. Absolute stacked bar charts were used to show the number of report-level stage-by-hallmark label associations across aging hallmark domains. Proportional stacked bar charts were used to display the relative distribution of functional, adaptive, and structural labels within each aging hallmark domain.

A co-occurrence network was generated to visualize relationships between mechanistic domains and aging hallmark domains. In the network, node size reflected label frequency, node color distinguished mechanistic and hallmark categories, and edge thickness reflected the frequency of co-occurrence between labels within the same report.

All graphical analyses were descriptive. They were intended to support evidence mapping and hypothesis generation rather than causal inference.

### 3.12 Quality appraisal, risk-of-bias considerations, and certainty assessment

Given the heterogeneity of included report types, a single formal risk-of-bias tool was not applied across the entire dataset. However, heterogeneity was not treated as a reason to omit quality consideration. Instead, we conducted a structured quality-appraisal summary stratified by major evidence category. Primary human studies, animal studies, cellular or mechanistic studies, systematic reviews, narrative reviews, and conceptual or theoretical papers were considered separately. For primary empirical studies, appraisal focused on study design, population or model relevance, exposure and outcome definition, mechanistic specificity, confounding or experimental control, and directness to the mapped bioenergetic–hallmark relationship. For systematic reviews, appraisal focused on search transparency, inclusion criteria, synthesis method, and whether risk-of-bias assessment was reported. For narrative reviews and conceptual papers, appraisal focused on source transparency, balance of evidence, risk of selective citation, dependence on recycled field narratives, and whether claims were grounded in primary evidence.

This quality-appraisal summary was used to contextualize interpretation rather than to exclude reports or generate pooled certainty estimates. It is provided in **Supplementary Table 3I**. The evidence-tiering framework was retained as a separate classification of inferential role and was not treated as a substitute for methodological quality appraisal, formal risk-of-bias assessment, reporting-bias assessment, or GRADE certainty grading. This category-level appraisal does not replace design-specific tools such as ROBINS-I, RoB 2, SYRCLE, QUADAS, AMSTAR 2, or GRADE. Because the present study was designed as an evidence map rather than an intervention-effect, diagnostic-accuracy, or prognosis review, the appraisal was used to contextualize evidentiary contribution rather than to generate pooled certainty ratings. Accordingly, the findings should not be interpreted as graded certainty-of-evidence conclusions.

### 3.13 Sensitivity and heterogeneity assessment

No formal statistical sensitivity analyses were conducted because the synthesis was descriptive and evidence-map based. However, we conducted a descriptive original-study-only sensitivity analysis to examine whether the main co-representation patterns remained visible after excluding reviews and conceptual papers. Robustness of observed patterns was considered qualitatively by comparing distributions across evidence type, biological model, hallmark domain, mechanistic domain, stage of impairment, and evidence tier.

Heterogeneity was explored descriptively rather than statistically. Sources of heterogeneity included study design, model system, population, disease context, mechanistic focus, measurement approach, and level of inference.

To address the heterogeneous composition of the evidence base and the inclusion of reviews and conceptual papers, we performed an original-study-only sensitivity analysis restricted to primary empirical reports, including human, animal, cellular, and mixed-model studies. Systematic reviews, narrative reviews, and conceptual papers were excluded from this subset. The purpose of this analysis was to evaluate whether the main mechanism–hallmark co-representation patterns remained visible when secondary and conceptual literature were removed.

For this subset, we recalculated mechanistic-domain frequencies, hallmark-domain frequencies, stage-label frequencies, biological-model distribution, evidence-tier distribution, and mechanism–hallmark co-occurrence counts, using the same report-level labeling approach as the main analysis. We also generated an original-study-only mechanism–hallmark co-occurrence network.

### 3.14 Data and materials availability

The full database search strategies are provided in **Supplementary Table 1**, and selected excluded reports with reasons are provided in **Supplementary Table 2**. The data extraction template, evidence-labeling framework, evidence-tiering framework, included-report characteristics, label matrix, co-occurrence matrix, figure-generation notes, data availability notes, and quality-appraisal summary by evidence category are provided in **Supplementary Table 3**.

## 4 Results

### 4.1 Study selection

**Figure 1:**
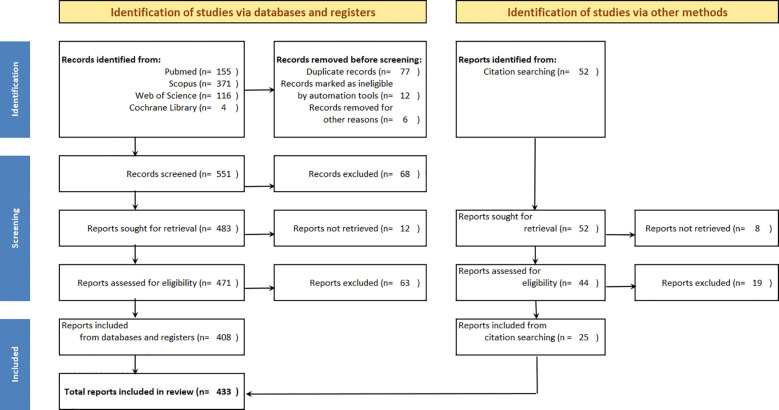
PRISMA 2020 flow diagram of study selection. Flow diagram showing identification, screening, retrieval, eligibility assessment, and inclusion of reports from database searches and citation searching. The final synthesis included 433 reports, comprising 408 reports from databases and registers and 25 reports identified through citation searching.

The study selection process is summarized in **Figure 1**. After screening, retrieval, and eligibility assessment, 408 reports from database searches and 25 reports from citation searching were included, yielding a final synthesis set of 433 reports. Consistent with PRISMA 2020 terminology, results are presented at the report level because formal study-level deduplication across all publication types was not performed. The final dataset was therefore treated as a structured evidence map of included reports rather than as a set of mutually exclusive primary studies.

### 4.2 Characteristics of the included evidence base

**Figure 2:**
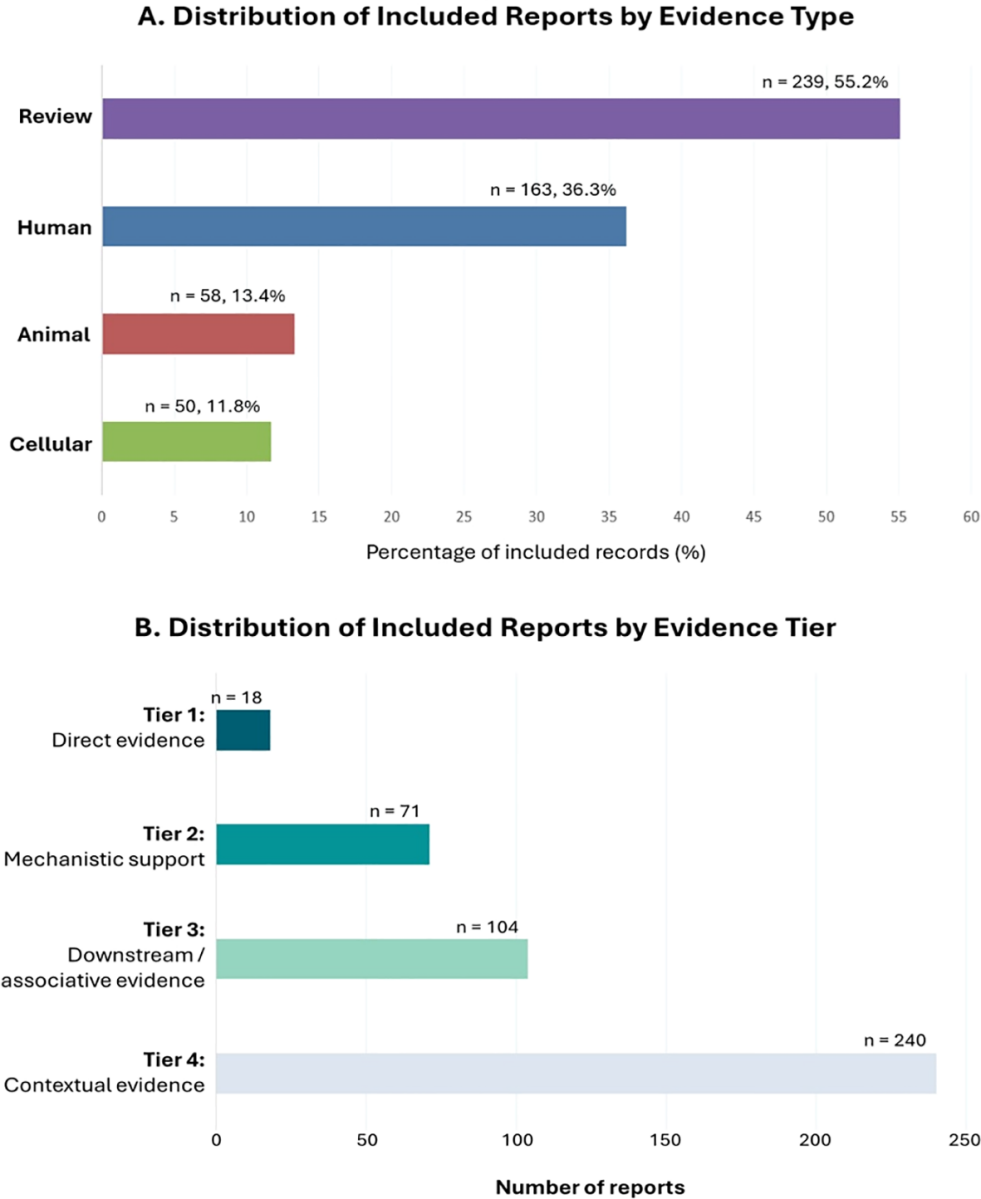
Overview of evidence-base composition and evidence-tier distribution. **(A)** Distribution of included reports by report-level evidence category. Categories were non-mutually exclusive; therefore, totals exceed the number of included reports. Percentages indicate the proportion of the full evidence map represented by each category. **(B)** Distribution of included reports by evidence tier. Tiers summarize the interpretive strength of evidence within the evidence map, ranging from direct evidence to contextual or conceptual evidence.

The included reports represented a heterogeneous evidence base spanning review-based, human, animal, and cellular literature. Review-based evidence was the largest category, followed by human, animal, and cellular evidence. **Table 1** summarizes the composition of the evidence base and the dominant evidence-map labels across evidence categories. **Figure 2** provides a visual overview of the evidence-base composition and evidence-tier distribution. Unless otherwise stated, all counts in the Results represent non-mutually exclusive report-level labels rather than mutually exclusive study totals; therefore, a single report could contribute to more than one evidence type, stage, mechanism, or hallmark domain.

Values are report-level counts. Evidence categories were non-mutually exclusive; therefore, human, animal, cellular, and review/conceptual categories may overlap and should not be summed across rows or columns. Original empirical reports were defined as reports not labeled as review, conceptual, or systematic review. Dominant labels indicate the most frequently assigned evidence-map labels within each category and do not imply causal direction, biological centrality, or mutually exclusive classification. MQC, mitochondrial quality control.

As shown in **Table 1**, 194 reports were classified as original empirical reports and 239 as review/conceptual reports. Human evidence was represented in 163 reports, animal evidence in 58 reports, and cellular evidence in 50 reports. Across the full evidence map and across major evidence categories, the dominant mechanistic labels were redox imbalance, substrate reallocation, oxidative throughput limitation, and ATP limitation, while the most frequent hallmark labels were altered intercellular communication, chronic inflammation, and deregulated nutrient sensing.

**Table 1:** Composition of the evidence base and dominant evidence-map labels by evidence category.

Evidence category	Original empirical	Review/conceptual	Dominant mechanistic labels	Dominant hallmark labels	Dominant stage labels
Full evidence map (433 reports)	194	239	Redox imbalance; substrate reallocation; oxidative throughput limitation	Altered intercellular communication; chronic inflammation; deregulated nutrient sensing	Adaptive; functional; structural
Human evidence (163 reports)	126	37	Redox imbalance; ATP limitation; substrate reallocation	Altered intercellular communication; chronic inflammation; deregulated nutrient sensing	Functional; adaptive; structural
Animal evidence (58 reports)	51	7	Redox imbalance; oxidative throughput limitation; ATP limitation	Altered intercellular communication; chronic inflammation; deregulated nutrient sensing	Adaptive; functional; structural
Cellular evidence (50 reports)	45	5	Oxidative throughput limitation; redox imbalance; ATP limitation; MQC impairment	Altered intercellular communication; chronic inflammation; deregulated nutrient sensing	Adaptive; functional; structural
Review/conceptual evidence (239 reports)	0	239	Redox imbalance; substrate reallocation; oxidative throughput limitation	Altered intercellular communication; chronic inflammation; deregulated nutrient sensing	Adaptive; functional; structural

This table summarizes the composition of the included evidence base by report-level evidence category. Evidence categories and evidence-map labels were non-mutually exclusive; therefore, counts should not be summed across rows or columns. Dominant labels represent the most frequently assigned labels within each category.

Evidence-tier classification showed fewer reports in the direct-evidence category and a larger contribution from mechanistic-support, downstream-association, and contextual-evidence categories (**Figure 2B**). A structured quality-appraisal summary further highlighted differences in the evidentiary contribution of included report types. Primary human studies provided the greatest translational relevance but varied in study design, population heterogeneity, biomarker selection, exposure and outcome definition, and ability to address confounding or temporality. Animal and cellular or mechanistic studies generally offered stronger mechanistic resolution and experimental control, but their directness to human aging and chronic disease contexts was more limited. Systematic reviews contributed structured synthesis where available, although their interpretive value depended on search transparency, inclusion criteria, synthesis method, and whether risk-of-bias assessment was reported. Narrative reviews and conceptual papers were useful for identifying field-level interpretations and proposed mechanisms, but were treated cautiously because of greater susceptibility to selective citation, narrative recycling, and dependence on secondary interpretation. To further assess whether the main descriptive patterns depended on secondary or conceptual literature, an original-study-only sensitivity analysis was conducted and is reported in Section 4.6, **Supplementary Table 4**, and **Supplementary Figure 1**. The quality-appraisal summary is provided in **Supplementary Table 3I**.

### 4.3 Distribution across aging hallmark domains

**Figure 3:**
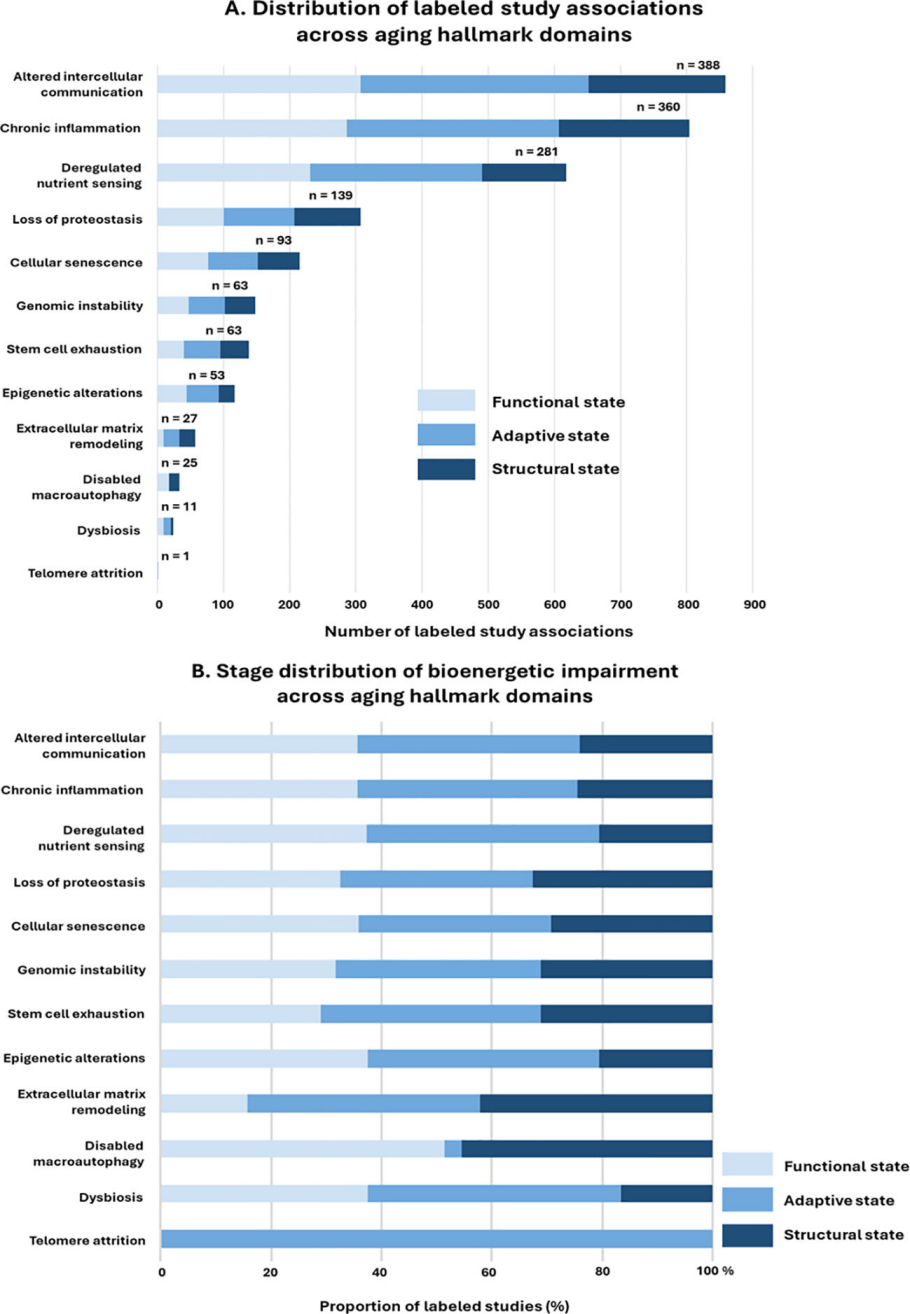
Distribution of functional, adaptive, and structural bioenergetic impairment labels across aging hallmark domains. **(A)** Absolute number of report-level stage-by-hallmark label associations across aging hallmark domains. **(B)** Proportional distribution of functional, adaptive, and structural labels within each hallmark domain. Counts represent report-level label associations assigned during evidence mapping, not repeated within-report mentions. Because labels were non-mutually exclusive, a single report could contribute to multiple hallmark domains and stage categories; therefore, totals represent labeled associations rather than mutually exclusive reports.

Within this mechanism-oriented dataset, labeled evidence was unevenly distributed across aging hallmark domains (**Figure 3A**); these distributions reflect visibility within the search frame rather than prevalence across the broader aging literature. The largest number of labeled associations involved altered intercellular communication, chronic inflammation, and deregulated nutrient sensing. Lower-frequency domains should be interpreted cautiously in light of small label counts and the original search strategy, which preferentially captured mitochondrial, metabolic, bioenergetic, inflammatory, and systemic-dysfunction terminology.

### 4.4 Stage distribution of bioenergetic impairment across hallmark domains

Stage-based mapping showed that most hallmark domains contained a mixture of functional, adaptive, and structural labels rather than aligning with a single stage of impairment (**Figure 3B**). Functional-stage labels reflected altered bioenergetic performance, adaptive-stage labels reflected compensatory or stress-responsive remodeling, and structural-stage labels reflected persistent organelle-, cellular-, or tissue-level disruption.

The proportional stage distribution varied across hallmark domains, with some domains showing relatively balanced representation and others showing greater weighting toward adaptive or structural labels. Because stage assignment was context-dependent, these distributions should be interpreted as descriptive evidence-map patterns rather than causal or temporal stage sequences. Ambiguous processes and coding decision rules are described in Section 3.8 and **Supplementary Table 3**.

### 4.5 Core mechanistic domains

**Figure 4:**
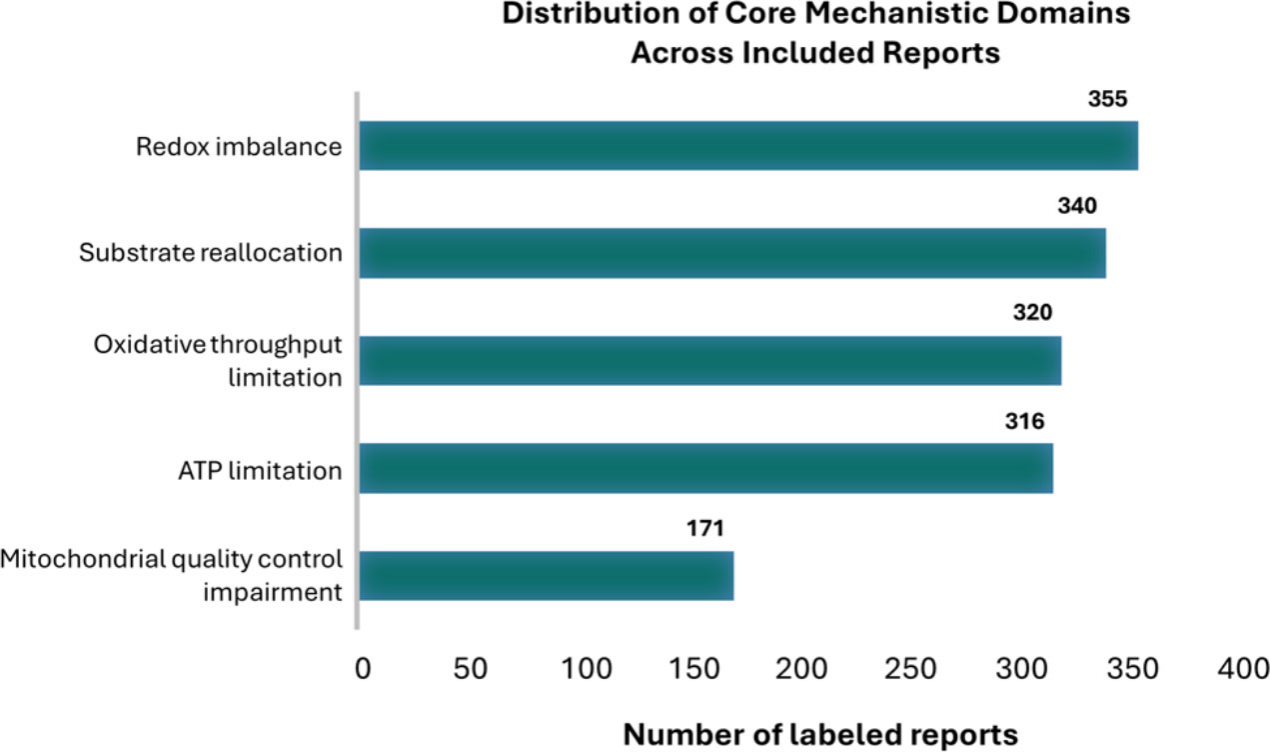
Distribution of core mechanistic domains across included reports. Horizontal bar chart showing the frequency of mechanistic labels assigned during evidence mapping. Redox imbalance, substrate reallocation, oxidative throughput limitation, and ATP limitation were the most frequently represented domains, while mitochondrial quality control impairment was less frequent but remained recurrent. Reports could receive multiple mechanistic labels; therefore, totals exceed the number of included reports.

Mechanistic labeling identified redox imbalance, substrate reallocation, oxidative throughput limitation, and ATP limitation as the most frequently assigned core mechanistic domains (**Figure 4**). Mitochondrial quality-control impairment was less frequently assigned but remained a recurrent label. These patterns indicate recurring mechanistic emphases across the included reports rather than independent prevalence estimates.

### 4.6 Co-occurrence and cross-domain co-labeling patterns

**Figure 5:**
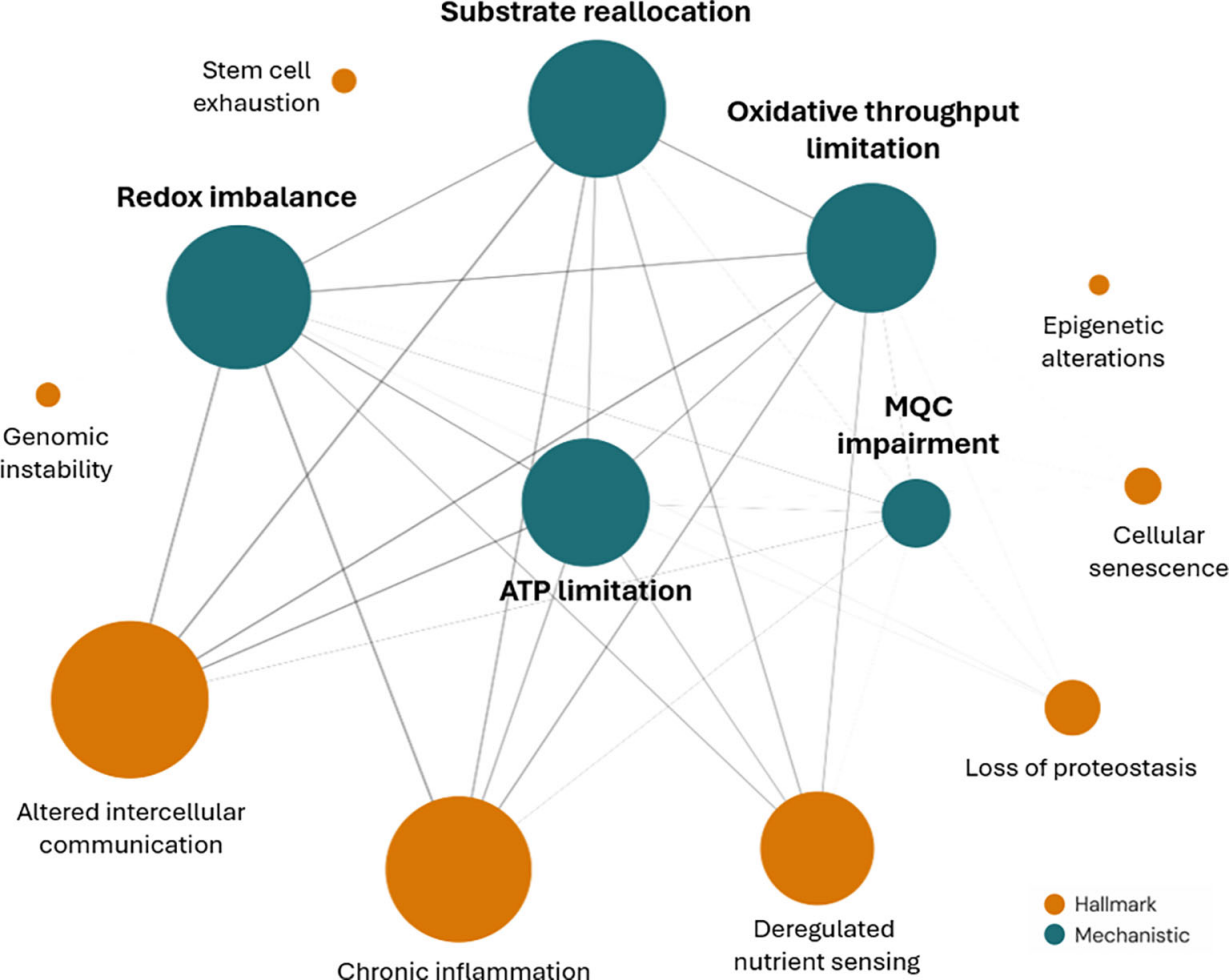
Co-occurrence network of mechanistic domains and aging hallmark domains. Network visualization showing report-level co-labeling between mechanistic domains and aging hallmark domains. Nodes represent assigned evidence-map labels. Edges represent reports in which two labels were assigned to the same report. Edge weight reflects the number of reports sharing that label combination. Labels were non-mutually exclusive, and repeated mentions within the same report were not counted multiple times. The network therefore represents patterns of co-representation within the mapped literature, not causal relationships, temporal ordering, mechanistic hierarchy, or biological centrality.

The co-occurrence network showed that core mechanistic labels were frequently co-assigned with multiple aging hallmark labels within the same reports (**Figure 5**). Redox imbalance, substrate reallocation, oxidative throughput limitation, and ATP limitation were among the most frequently co-labeled mechanistic domains and most often appeared alongside altered intercellular communication, chronic inflammation, and deregulated nutrient sensing. Node size and edge thickness indicate label frequency and co-labeling frequency only, not causal strength, biological importance, directionality, mechanistic hierarchy, or hub biology.

Because the main evidence map included original studies, reviews, and conceptual papers, we performed an original-study-only sensitivity analysis to examine whether the observed co-representation patterns persisted after excluding secondary and conceptual literature. Within this primary empirical subset, the most frequently assigned mechanistic labels were redox imbalance, ATP limitation, substrate reallocation, oxidative throughput limitation, and mitochondrial quality-control impairment. Redox imbalance was assigned to 161 primary reports, ATP limitation to 144, substrate reallocation to 126, oxidative throughput limitation to 120, and mitochondrial quality-control impairment to 74. The most frequently assigned hallmark labels were altered intercellular communication, chronic inflammation, and deregulated nutrient sensing, appearing in 165, 156, and 108 primary reports, respectively.

Stage-based labels also remained distributed across functional, adaptive, and structural categories. Functional-stage labels were assigned to 168 primary reports, adaptive-stage labels to 155, and structural-stage labels to 83. This pattern indicates that functional and adaptive bioenergetic labels were especially visible in the primary empirical literature, whereas structural labels were less frequent but still recurrent.

The original-study-only mechanism–hallmark co-occurrence matrix and network are provided in **Supplementary Table 4A** and **Supplementary Figure 1**. The network showed continued co-representation between core bioenergetic mechanisms and regulatory or systems-level hallmark domains, particularly altered intercellular communication, chronic inflammation, and deregulated nutrient sensing. However, these findings remain descriptive. They indicate that the main co-representation pattern was not solely driven by reviews or conceptual papers.

### 4.7 Distributional imbalances and areas of lower visibility

Several areas of lower visibility were identified within the mechanism-oriented evidence map. Some hallmark domains were sparsely represented, particularly telomere attrition, dysbiosis, extracellular matrix remodeling, and selected molecular damage-related domains. Some mitochondrial regulatory mechanisms, including mitochondrial dynamics and calcium signaling, were less prominent than redox-, substrate-, ATP-, and throughput-related labels.

Relatively few reports directly examined temporal transitions across functional, adaptive, and structural stages of impairment. Longitudinal human studies and intervention studies explicitly testing bioenergetic recovery, resilience, or stage reversal were also limited.

### 4.8 Summary of findings

In summary, the included reports formed a broad but heterogeneous mechanism-enriched evidence base derived from a previously curated search strategy. Label distributions were concentrated in regulatory and systems-level hallmark domains, particularly altered intercellular communication, chronic inflammation, and deregulated nutrient sensing, whereas several molecular and structural domains were less visible within this mechanism-oriented search frame. This lower visibility should not be interpreted as weaker biological relevance. Core bioenergetic mechanisms were recurrently represented, and stage-based mapping showed frequent coexistence of functional, adaptive, and structural labels. These findings provide the descriptive basis for the interpretive synthesis developed in the Discussion.

## 5 Discussion

### 5.1 Principal findings

This secondary evidence map synthesized 433 reports from a previously curated, mechanism-oriented dataset and should not be interpreted as a comprehensive hallmark-by-hallmark review of the aging literature. The Discussion separates three levels of interpretation: report-level labels that were counted, recurring label distributions and co-labeling patterns that were observed, and the bioenergetic constraint or mitochondrial congestion model proposed as a hypothesis-generating interpretation. The model was not directly tested by the present analysis.

Three principal findings emerged. First, mapped evidence was concentrated in regulatory and systems-level hallmarks, particularly altered intercellular communication, chronic inflammation, and deregulated nutrient sensing. This distribution is consistent with contemporary aging frameworks in which mitochondrial dysfunction is not viewed as an isolated hallmark, but as a process that interacts with nutrient-sensing pathways, inflammatory signaling, cellular communication, and systemic regulation (López-Otín et al., 2013, 2023). However, this concentration should be interpreted as a pattern of visible co-representation within the curated dataset, not as evidence that these domains are intrinsically more bioenergetically important than lower-frequency hallmarks.

Second, mechanistic labeling showed recurrent co-representation around redox imbalance, substrate reallocation, oxidative throughput limitation, and ATP limitation. These domains align with evidence that mitochondria integrate energetic, redox, inflammatory, and stress-related signals, thereby influencing both cellular function and organism-level adaptation (Picard et al., 2018; Xu et al., 2025).

Third, stage-based mapping indicated that functional, adaptive, and structural labels frequently coexisted across hallmark domains. This pattern supports the potential usefulness of a dynamic continuum as an organizing interpretation, rather than treating mitochondrial and bioenergetic impairment as a single static category. In this framework, early functional constraint may coexist with compensatory adaptation and, in some contexts, with downstream structural deterioration. However, because the included evidence was broad and heterogeneous, with substantial review-based and contextual literature, these findings should be interpreted as evidence-map convergence rather than proof of a definitive causal hierarchy.

Together, these findings support framework-building and hypothesis generation. Bioenergetic constraint may provide a useful organizing lens for interpreting recurrent co-representation across selected hallmark domains within this evidence map, but causal direction, temporal sequence, and clinical applicability require further testing in longitudinal, interventional, and mechanistically specific studies.

### 5.2 Frequently represented hallmark domains within the mechanism-oriented evidence map

A prominent finding was the concentration of mapped evidence within three regulatory and systems-level domains: altered intercellular communication, chronic inflammation, and deregulated nutrient sensing. These domains were the most frequently represented hallmark labels within the evidence map, suggesting that mitochondrial and bioenergetic mechanisms were most visibly co-represented with signaling, inflammatory, and metabolic regulatory processes. This interpretation is biologically plausible because these domains are often discussed as interfaces through which mitochondrial bioenergetic states may be translated into broader cellular, tissue-level, and systemic responses.

The prominence of deregulated nutrient sensing is consistent with the role of nutrient-sensing pathways in coordinating substrate availability, ATP demand, redox status, mitochondrial activity, autophagy, proteostasis, and cellular stress responses. Pathways such as insulin/IGF-1 signaling, mTOR, AMPK, and sirtuin-related regulation link nutrient status to mitochondrial function and downstream decisions about maintenance, repair, growth, and stress adaptation (López-Otín et al., 2013, 2023). Although the present evidence map does not establish directionality, the frequent representation of nutrient-sensing domains supports the interpretation that these pathways may serve as important interfaces between mitochondrial bioenergetic adaptation and broader aging-related processes.

Chronic inflammation and altered intercellular communication may represent related layers of mitochondrial signal propagation. Mitochondria are increasingly understood as dynamic signaling and information-processing organelles that integrate metabolic and environmental inputs and generate outputs influencing nuclear gene expression, immune signaling, intercellular communication, and organismal adaptation (Picard and Shirihai, 2022). Under conditions of bioenergetic strain, mitochondrial stress signals may contribute to inflammatory tone and immune activation, while inflammatory mediators can feed back onto mitochondrial metabolism, substrate allocation, and energy demand (Xu et al., 2025; Gorgori-Gonzalez et al., 2026). Similarly, mitochondrial stress or energetic limitation may be communicated across cells and tissues through cytokine, endocrine, vesicular, metabolic, or stress-response pathways, making mitochondrial state visible beyond the organelle or individual cell (Bar‐Ziv et al., 2020; Bilen et al., 2022; Burtscher et al., 2023; Wang and Zhang, 2025; Zhang et al., 2025).

### 5.3 Interpreting mapped mitochondrial dysfunction through a staged bioenergetic continuum

The coexistence of functional, adaptive, and structural labels across hallmark domains supports the value of interpreting mitochondrial impairment as a dynamic continuum rather than a binary state of “function” versus “dysfunction.” This directly addresses a central problem in the literature: the term “mitochondrial dysfunction” often compresses reversible functional constraint, compensatory remodeling, and more persistent structural disruption into a single category. This interpretation is consistent with calls for more precise mitochondrial terminology that distinguishes among mitochondrial properties, activities, functions, and behaviors rather than treating mitochondrial change as uniformly pathological (Monzel et al., 2023).

A staged bioenergetic continuum may therefore provide a more precise organizing framework. At the functional level, mitochondrial impairment may be reflected by reduced oxidative capacity, impaired respiratory reserve, redox imbalance, oxidative throughput limitation, or ATP limitation. These changes describe constraints in energy conversion and redox handling before irreversible structural damage is necessarily present. At the adaptive level, cells and tissues may respond through substrate reallocation, glycolytic shift, altered nutrient sensing, inflammatory signaling, and modified intercellular communication (Bar‐Ziv et al., 2020; Boardman et al., 2023; Burtscher et al., 2023). These responses may initially serve compensatory or protective roles by redistributing substrates, limiting excess energetic demand, or preserving short-term viability under stress (Tippairote et al., 2022). At the structural level, reports describing persistent or unresolved bioenergetic strain often also described mitochondrial quality-control impairment, organelle remodeling, senescence, loss of proteostasis, extracellular matrix remodeling, stem-cell exhaustion, or tissue degeneration (Roca-Portoles and Tait, 2021; Gorgori-Gonzalez et al., 2026; Tsui et al., 2026). This staged interpretation is consistent with broader views of mitochondria as dynamic signaling and adaptation systems rather than static indicators of cellular damage (Picard and Shirihai, 2022).

The value of this continuum is conceptual precision. It separates reversible functional constraint, compensatory remodeling, and more persistent structural disruption—processes that are often compressed into the single phrase “mitochondrial dysfunction.” Importantly, the observed co-occurrence of stage labels supports conceptual organization rather than a confirmed temporal sequence. These features may occur simultaneously, vary across tissues, and differ according to stress duration, substrate availability, inflammatory context, disease state, and organismal reserve. The staged model should therefore be understood as an organizing framework for clarifying heterogeneous mitochondrial findings, not as proof of universal progression.

### 5.4 Hypothesis-generating interpretation of recurrently co-labeled mechanisms

The mechanistic-domain analysis counted report-level labels assigned to redox imbalance, substrate reallocation, oxidative throughput limitation, ATP limitation, and mitochondrial quality-control impairment. These labels were frequently assigned within the same evidence base and often co-occurred with regulatory and systems-level hallmark labels. The following synthesis therefore presents one hypothesis-generating interpretation of these co-labeling patterns: that redox disturbance, constrained oxidative processing, ATP limitation, substrate rerouting, and mitochondrial quality-control impairment may interact under some aging-related conditions.

Redox imbalance and reductive pressure may be one possible interpretive entry point into this coupled process. Under chronic stress, inflammation, disease, aging, or repeated energetic demand, substrate mobilization may increase to support adaptive responses. However, when substrate-derived reducing equivalents exceed mitochondrial electron transport-chain processing capacity, NADH and FADH₂ may accumulate, creating reductive pressure and limiting NAD^+^ regeneration. A more reduced NADH/NAD^+^ state may slow dehydrogenase-dependent reactions, restrict tricarboxylic acid cycle flux, impair fatty acid oxidation, and increase electron leakage (Muoio, 2014; Borkum, 2023). Included reports support this interpretation across different contexts, including type 2 diabetes, where platelet mitochondria showed diminished oxygen consumption, reduced oxygen-dependent ATP synthesis, and antioxidant stress responses, and aged retinal pigment epithelial cells, where reduced mitochondrial ATP production and respiratory reserve were associated with increased oxidative-stress susceptibility (Avila et al., 2012; Rohrer et al., 2016). These findings are consistent with redox imbalance functioning both as a consequence of impaired electron handling and as a contributor to further oxidative constraint.

**Figure 6:**
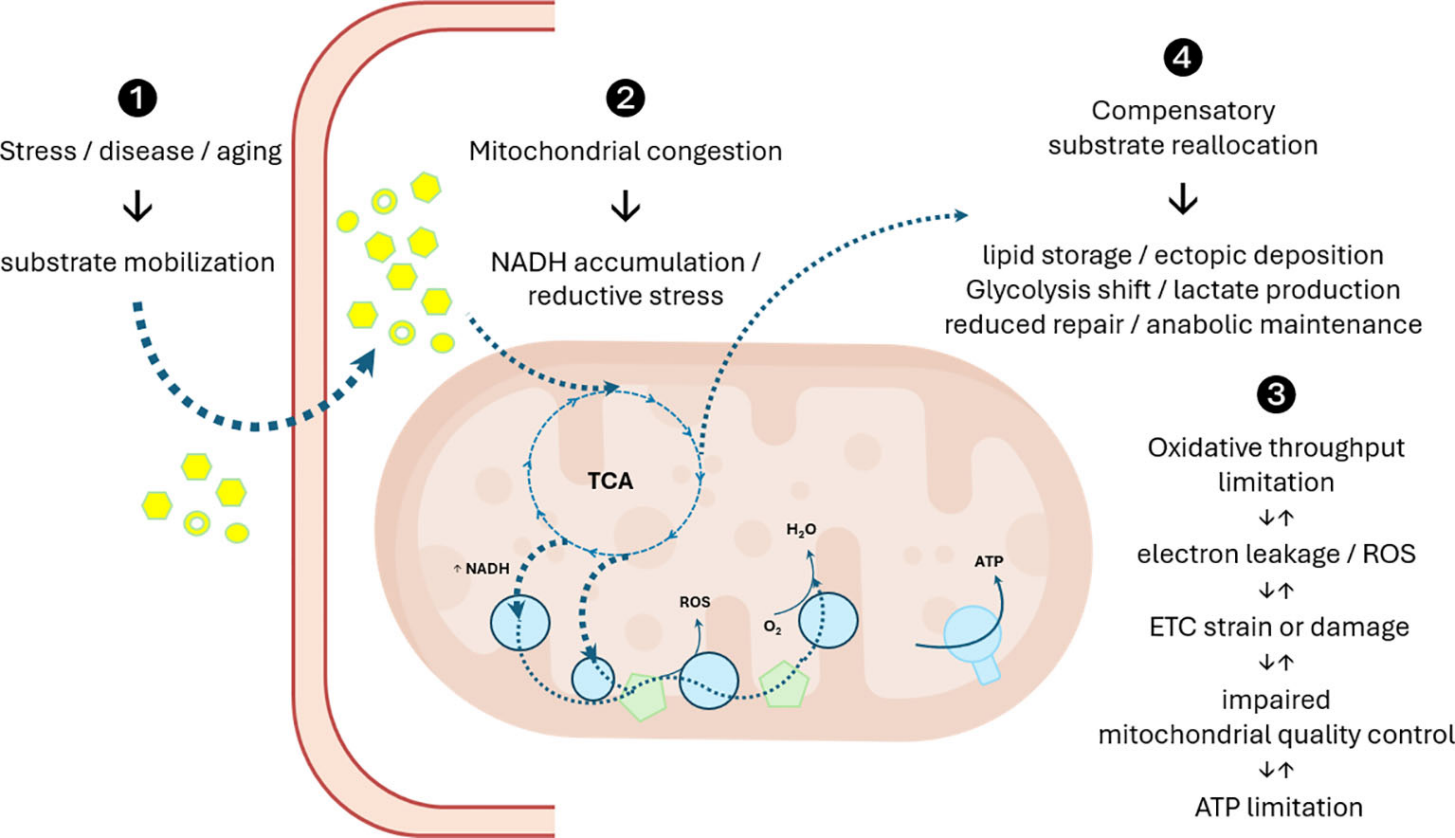
Proposed mitochondrial congestion model linking substrate mobilization to bioenergetic constraint. Conceptual schematic showing how chronic stress, disease, inflammation, aging, or repeated energetic demand may increase substrate-derived reducing-equivalent load. When this load exceeds mitochondrial electron transport-chain processing capacity, functional congestion may develop, contributing to redox disturbance, constrained oxidative throughput, ATP limitation, impaired mitochondrial renewal, and compensatory substrate rerouting. The model is not derived from the co-occurrence network and does not represent a tested causal pathway; it summarizes a proposed interpretive model generated from recurrent co-labeling patterns and prior mitochondrial biology.

Oxidative throughput limitation may provide one possible interpretive concept for relating these observations, although the present evidence map cannot determine whether impaired throughput is upstream, downstream, compensatory, or tissue-specific in any given context. The key issue is not simply whether mitochondria are “functional” or “damaged,” but whether they can convert incoming substrates and reducing equivalents into usable ATP while preserving redox balance, respiratory efficiency, and electron transport-chain integrity. In contexts where oxidative processing capacity is constrained, substrate pressure and redox disturbance may reinforce one another, progressively narrowing effective oxidative capacity. In an elderly population cohort, central obesity indices were associated with reduced basal respiration, maximal respiration, spare respiratory capacity, and ATP production in peripheral blood mononuclear cells, suggesting that metabolic risk may coincide with impaired cellular bioenergetic throughput (Attachaipanich et al., 2025). Plasma proteomic signatures associated with skeletal muscle oxidative capacity were also enriched in pathways related to energy metabolism, proteostasis, oxidative stress responses, and inflammation (Zampino et al., 2020). These findings are consistent with the hypothesis that oxidative throughput limitation may contribute to bioenergetic constraint in some aging-related contexts. This proposed relationship is summarized conceptually in **Figure 6**.

ATP limitation may represent one functional manifestation of constrained throughput. When electron flow, redox balance, and oxidative phosphorylation become inefficient, ATP availability and respiratory reserve may decline. This matters because many aging-related maintenance processes are energy-dependent, including proteostasis, autophagy, mitophagy, ion transport, DNA repair, immune regulation, tissue remodeling, and adaptive stress responses. In skeletal muscle aging, mitochondrial quality control and the autophagy–lysosome pathway have been described as important for preserving myocyte viability and removing damaged proteins and organelles (Picca et al., 2023; Marzetti et al., 2025). In older gastric cancer patients with cachexia, altered expression of mitochondrial fusion, fission, and mitophagy-related mediators further suggests disruption of mitochondrial renewal pathways during systemic energetic stress and tissue wasting (Marzetti et al., 2017). ATP limitation may therefore reflect not only impaired energy production, but also reduced capacity for repair, renewal, and adaptive recovery.

Substrate reallocation may be interpreted as a compensatory response in some constrained bioenergetic states. When mitochondrial oxidative throughput is insufficient to fully process mobilized substrates, cells may redirect fuel toward glycolysis, lactate production, lipid storage, amino acid catabolism, or biosynthetic pathways. This rerouting may help maintain ATP production, regenerate NAD^+^, buffer substrate excess, or support short-term stress responses; however, if sustained, it may also indicate that substrate availability has exceeded oxidative processing capacity. In Fanconi anemia cells, impaired oxidative phosphorylation was accompanied by increased glycolytic flux, with glycolysis supporting energy production and helping balance the NADH/NAD^+^ ratio through pyruvate-to-lactate conversion (Cappelli et al., 2017). In senescent cells, metabolic reprogramming has been characterized by increased glycolysis, altered fatty acid oxidation, lipid droplet accumulation, and reduced mitochondrial oxidative phosphorylation (Liu et al., 2023). These examples support the interpretation of substrate reallocation as adaptive rerouting under bioenergetic constraint rather than simply as metabolic flexibility.

Mitochondrial quality-control impairment could plausibly interact with bioenergetic constraint, although directionality cannot be inferred here. Persistent redox disturbance, ATP limitation, and substrate overload may compromise mitophagy, mitochondrial biogenesis, fusion–fission dynamics, and organelle turnover. Conversely, impaired mitochondrial quality control may reduce respiratory capacity, worsen electron leakage, and further constrain oxidative throughput. In aging muscle, mitochondrial quality-control pathways are closely linked to inflammatory, mitochondrial, and senescence-related processes (Picca et al., 2023). Earlier work also proposed that damaged mitochondria may accumulate through oxidative injury, impaired autophagic degradation, lysosomal lipofuscin loading, and reduced proteolytic efficiency (Terman and Brunk, 2004). Thus, impaired mitochondrial renewal may act as both a consequence and amplifier of bioenergetic constraint.

Taken together, these co-labeling patterns are compatible with, but do not test, a proposed mitochondrial congestion model in which substrate-derived reducing-equivalent load may exceed oxidative processing capacity under some conditions of chronic stress, inflammation, disease, aging, or repeated energetic demand. In this model, redox disturbance, constrained oxidative throughput, ATP limitation, impaired mitochondrial renewal, and compensatory substrate rerouting are proposed as potentially interacting features of bioenergetic constraint. The present evidence map cannot determine whether these features are upstream, downstream, compensatory, parallel, tissue-specific, or context-dependent. The model should therefore be treated as hypothesis-generating.

Overall, the evidence map supports the visibility of recurring mechanistic labels, whereas the bioenergetic constraint model remains an interpretive proposal about how these labels may relate biologically.

### 5.5 Limitations, boundary conditions, and future directions

The main limitation is that the original search strategy shaped the evidence map; therefore, the results should not be interpreted as a comprehensive or proportional map of the full aging-hallmark literature. Because the dataset was generated through a mechanism-oriented search, it preferentially captured literature in which mitochondrial, metabolic, bioenergetic, inflammatory, senescence-related, or stress-adaptation mechanisms were explicit. Sparse representation of telomere attrition, extracellular matrix remodeling, dysbiosis, genomic instability, epigenetic alterations, or other molecular and structural domains should therefore be interpreted as limited visibility within this search frame. Conversely, their high representation reflects this curated dataset and should not be interpreted as proof of greater biological centrality.

Underrepresented domains may also reflect differences in terminology, indexing, research density, and disciplinary framing. For example, mitochondrial redox state, oxidative stress, substrate availability, and metabolic stress may intersect with DNA damage responses, telomere dynamics, and epigenetic regulation (Epel, 2009; Fernandes et al., 2021; Gürel et al., 2024; Monti et al., 2025). Similarly, stem-cell exhaustion and tissue remodeling may involve mitochondrial metabolism, inflammatory signaling, and energetic state, even when these processes are described through the language of regeneration, niche biology, matrix remodeling, or senescence rather than bioenergetic constraint (Marinelli Busilacchi et al., 2024; Xu et al., 2024; Liu et al., 2025).

Another boundary condition is directionality. Co-labeling cannot determine whether bioenergetic constraint precedes, follows, amplifies, or compensates for other aging processes. Similarly, the staged framework should be read as an organizing model rather than evidence of a fixed biological sequence. This caution is consistent with broader evidence that mitochondrial biology interacts bidirectionally with inflammation, nutrient sensing, intercellular communication, senescence-associated pathways, and tissue remodeling (Hotamisligil and Erbay, 2008; Franceschi et al., 2018; Chi, 2022; Picca et al., 2023; Zhang et al., 2025). A further boundary condition concerns the network visualization itself. **Figure 5** was generated from non-mutually exclusive report-level labels and therefore visualizes co-labeling frequency only. It should not be interpreted as a biological network, pathway model, causal graph, or evidence of hub biology.

The original-study-only sensitivity analysis partly addressed the concern that the main evidence map could be shaped by secondary and conceptual literature. After excluding reviews and conceptual papers, the primary empirical subset retained a broadly similar descriptive pattern, with redox imbalance, ATP limitation, substrate reallocation, oxidative throughput limitation, and mitochondrial quality-control impairment co-represented with several aging hallmark domains. However, this analysis remains descriptive and subject to the same constraints of mechanism-oriented searching, heterogeneous study designs, and report-level co-labeling.

A related limitation is that this review did not perform design-specific formal risk-of-bias assessment for every included report. To address this limitation, we added a structured quality-appraisal summary by evidence category in **Supplementary Table 3I**. This appraisal should be interpreted as contextual rather than equivalent to formal GRADE certainty assessment or design-specific risk-of-bias evaluation. The evidence-tiering framework classifies inferential role within the map, but it does not establish methodological quality or certainty of evidence.

A further limitation is that the functional–adaptive–structural framework required interpretive classification. Although operational decision rules, consensus checking, adjudication of unresolved cases, and representative ambiguous-case examples were used to reduce circularity, formal inter-rater agreement statistics were not calculated. The staging scheme should therefore not be interpreted as a validated biological staging system. Some processes, including inflammation, nutrient sensing, mitophagy, mitochondrial quality control, and senescence, may function differently depending on tissue, stress duration, disease context, and measurement approach. The stage labels identify how these processes were represented within each report’s mechanistic narrative, rather than assigning fixed biological identities or causal positions to the processes themselves.

Future studies should therefore move from evidence mapping toward direct testing of stage transitions and mechanistic directionality. Priority areas include longitudinal human studies measuring bioenergetic function before downstream hallmark changes emerge; tissue-specific studies comparing mitochondrial mechanisms across muscle, immune, brain, adipose, gut, and vascular systems; interventional studies testing whether restoration of oxidative throughput, redox balance, ATP reserve, or mitochondrial quality control modifies aging-related outcomes; and experimental models designed to distinguish functional limitation, adaptive remodeling, and structural impairment over time. Future studies should also evaluate whether bioenergetic markers can serve as resilience metrics, including recovery of oxidative capacity, ATP reserve, redox balance, mitochondrial quality control, inflammatory resolution, and functional performance after standardized physiological challenges.

## 6 Conclusion

This secondary evidence map identified recurrent co-representation of mitochondrial and bioenergetic mechanisms with several aging hallmark domains visible within a previously curated, mechanism-oriented literature set. The strongest mapped representation involved regulatory and systems-level domains, particularly chronic inflammation, altered intercellular communication, and deregulated nutrient sensing. Core mechanisms related to redox balance, substrate handling, oxidative processing, ATP availability, and mitochondrial quality control were repeatedly co-labeled with these domains, supporting bioenergetic constraint as a cautious interpretive lens for examining how metabolic state, stress signaling, inflammatory tone, cellular communication, and maintenance or repair capacity are discussed together in the mapped literature.

Stage-based mapping further supports the usefulness of distinguishing functional limitation, adaptive remodeling, and structural impairment within aging-related mitochondrial dysfunction; however, the broader concept of bioenergetic constraint remains an interpretive framework derived from these mapped features rather than a directly tested mechanism. This distinction may help move interpretation beyond a binary view of mitochondrial function versus dysfunction and toward a more dynamic framework in which bioenergetic constraint can be examined in relation to different phases of adaptation and decline.

These conclusions remain cautious and should not be generalized to the full aging-hallmark literature without further hallmark-specific searches and primary empirical testing. Longitudinal, experimental, tissue-specific, and interventional studies are needed to determine whether early bioenergetic limitation is temporally associated with hallmark progression, impaired resilience, and aging-related outcomes, and whether restoring oxidative throughput, redox balance, ATP reserve, or mitochondrial quality control can modify these trajectories.

## Data Availability

The original contributions presented in the study are included in the article/**Supplementary Material**. Further inquiries can be directed to the corresponding author.

## References

[B1] Attachaipanich T. Sriwichaiin S. Apaijai N. Thanyaratsarun T. Thongmung N. Vathesatogkit P. . (2025). Obesity classified by anthropometric parameters was associated with mitochondrial bioenergetics impairment of peripheral blood mononuclear cells in the elderly population. Exp. Gerontology 202, 112724. doi: 10.1016/j.exger.2025.112724 40037474

[B2] Avila C. Huang R. J. Stevens M. V. Aponte A. M. Tripodi D. Kim K. Y. . (2012). Platelet mitochondrial dysfunction is evident in type 2 diabetes in association with modifications of mitochondrial anti-oxidant stress proteins. Exp. Clin. Endocrinol. Diabetes Off. Journal German Soc. Endocrinol. [and] German Diabetes Assoc. 120, 248–251. doi: 10.1055/s-0031-1285833 21922457 PMC6122851

[B3] Bar-Ziv R. Bolas T. Dillin A. (2020). Systemic effects of mitochondrial stress. EMBO Rep. 21, e50094. doi: 10.15252/embr.202050094 32449292 PMC7271648

[B4] Bilen M. Benhammouda S. Slack R. S. Germain M. (2022). The integrated stress response as a key pathway downstream of mitochondrial dysfunction. Curr. Opin. Physiol. 27, 100555. doi: 10.1016/j.cophys.2022.100555 38826717

[B5] Boardman N. T. Trani G. Scalabrin M. Romanello V. Wüst R. C. I. (2023). Intracellular to interorgan mitochondrial communication in striated muscle in health and disease. Endocr. Rev. 44, 668–692. doi: 10.1210/endrev/bnad004 36725366 PMC10335175

[B6] Borkum J. M. (2023). The tricarboxylic acid cycle as a central regulator of the rate of aging: Implications for metabolic interventions. Adv. Biol. 7, 2300095. doi: 10.1002/adbi.202300095 37132059

[B7] Brand M. D. Nicholls D. G. (2011). Assessing mitochondrial dysfunction in cells. Biochem. J. 435, 297–312. doi: 10.1042/bj20110162 21726199 PMC3076726

[B8] Burtscher J. Soltany A. Visavadiya N. P. Burtscher M. Millet G. P. Khoramipour K. . (2023). Mitochondrial stress and mitokines in aging. Aging Cell n/a, e13770. doi: 10.1111/acel.13770 36642986 PMC9924952

[B9] Calvo S. E. Mootha V. K. (2010). The mitochondrial proteome and human disease. Annu. Rev. Genomics Hum. Genet. 11, 25–44. doi: 10.1146/annurev-genom-082509-141720 20690818 PMC4397899

[B10] Cappelli E. Cuccarolo P. Stroppiana G. Miano M. Bottega R. Cossu V. . (2017). Defects in mitochondrial energetic function compels Fanconi anaemia cells to glycolytic metabolism. Biochim. Biophys. Acta (BBA) - Mol. Basis Dis. 1863, 1214–1221. doi: 10.1016/j.bbadis.2017.03.008 28315453

[B11] Chi H. (2022). Immunometabolism at the intersection of metabolic signaling, cell fate, and systems immunology. Cell. Mol. Immunol. 19, 299–302. doi: 10.1038/s41423-022-00840-x 35190684 PMC8891332

[B12] Cohen P. (2014). New role for the mitochondrial peptide humanin: Protective agent against chemotherapy-induced side effects. J. Natl. Cancer Inst. 106, dju006. doi: 10.1093/jnci/dju006 24586106 PMC3982780

[B13] Epel E. S. (2009). Psychological and metabolic stress: A recipe for accelerated cellular aging? Hormones 8, 7–22. doi: 10.14310/horm.2002.1217 19269917

[B14] Fernandes S. G. Dsouza R. Khattar E. (2021). External environmental agents influence telomere length and telomerase activity by modulating internal cellular processes: Implications in human aging. Environ. Toxicol. Pharmacol. 85, 103633. doi: 10.1016/j.etap.2021.103633 33711516

[B15] Franceschi C. Garagnani P. Parini P. Giuliani C. Santoro A. (2018). Inflammaging: A new immune–metabolic viewpoint for age-related diseases. Nat. Rev. Endocrinol. 14, 576–590. doi: 10.1038/s41574-018-0059-4 30046148

[B16] Gorgori-Gonzalez A. Soto-Rodriguez S. Tamayo-Torres E. Garcia-Dominguez E. Sebastia V. Gambini J. . (2026). Leveraging mitochondrial stress to improve healthy aging. Sports Med. Health Sci. 8, 23–33. doi: 10.1016/j.smhs.2025.10.003 41646179 PMC12869046

[B17] Gürel S. Pak E. N. Tek N. A. (2024). Aging processes are affected by energy balance: Focused on the effects of nutrition and physical activity on telomere length. Curr. Nutr. Rep. 13, 264–279. doi: 10.1007/s13668-024-00529-9 38498288 PMC11133118

[B18] Hotamisligil G. S. Erbay E. (2008). Nutrient sensing and inflammation in metabolic diseases. Nat. Rev. Immunol. 8, 923–949. doi: 10.1038/nri2449 19029988 PMC2814543

[B19] Kroemer G. Maier A. B. Cuervo A. M. Gladyshev V. N. Ferrucci L. Gorbunova V. . (2025). From geroscience to precision geromedicine: Understanding and managing aging. Cell. 188, 2043–2062. doi: 10.1016/j.cell.2025.03.011 40250404 PMC12037106

[B20] Liu B. Meng Q. Gao X. Sun H. Xu Z. Wang Y. . (2023). Lipid and glucose metabolism in senescence. Front. Nutr. 10, 1157352. doi: 10.3389/fnut.2023.1157352 37680899 PMC10481967

[B21] Liu H. Wang S. Wang J. Guo X. Song Y. Fu K. . (2025). Energy metabolism in health and diseases. Signal. Transduct Target Ther. 10, 69. doi: 10.1038/s41392-025-02141-x 39966374 PMC11836267

[B22] López-Otín C. Blasco M. A. Partridge L. Serrano M. Kroemer G. (2013). The hallmarks of aging. Cell. 153, 1194–1217. doi: 10.1016/j.cell.2013.05.039 23746838 PMC3836174

[B23] López-Otín C. Blasco M. A. Partridge L. Serrano M. Kroemer G (2023). Hallmarks of aging: An expanding universe. Cell. 186(2), 243–278. doi: 10.1016/j.cell.2022.11.001 36599349

[B24] Marinelli Busilacchi E. Morsia E. Poloni A. (2024). Bone marrow adipose tissue. Cells 13, 724. doi: 10.3390/cells13090724 38727260 PMC11083575

[B25] Marzetti E. Di Lorenzo R. Picca A. (2025). The mitochondrial side of frailty. Curr. Opin. Clin. Nutr. Metab. Care 29, 224–230. doi: 10.1097/mco.0000000000001175 41076735

[B26] Marzetti E. Lorenzi M. Landi F. Picca A. Rosa F. Tanganelli F. . (2017). Altered mitochondrial quality control signaling in muscle of old gastric cancer patients with cachexia. Exp. Gerontology 87, 92–99. doi: 10.1016/j.exger.2016.10.003 27847330

[B27] McEwen B. S. (1998). Stress, adaptation, and disease. Allostasis and allostatic load. Ann. N. Y. Acad. Sci. 840, 33–44. doi: 10.1111/j.1749-6632.1998.tb09546.x 9629234

[B28] McEwen B. S. Wingfield J. C. (2003). The concept of allostasis in biology and biomedicine. Horm. Behav. 43, 2–15. doi: 10.1016/S0018-506X(02)00024-7 12614627

[B29] Mochly-Rosen D. Das K. Grimes K. V. (2012). Protein kinase C, an elusive therapeutic target? Nat. Rev. Drug Discov. 11, 937–957. doi: 10.1038/nrd3871 23197040 PMC3760692

[B30] Monti P. Biganzoli E. Bollati V. (2025). Impact of air pollution and occupational inhalation exposures on neurodegenerative disorders: An epigenetic perspective. iScience 28(7), 112825. doi: 10.1016/j.isci.2025.112825 40599315 PMC12209981

[B31] Monzel A. S. Enríquez J. A. Picard M. (2023). Multifaceted mitochondria: Moving mitochondrial science beyond function and dysfunction. Nat. Metab. 5, 546–562. doi: 10.1038/s42255-023-00783-1 37100996 PMC10427836

[B32] Mootha V. K. Lindgren C. M. Eriksson K.-F. Subramanian A. Sihag S. Lehar J. . (2003). PGC-1α-responsive genes involved in oxidative phosphorylation are coordinately downregulated in human diabetes. Nat. Genet. 34, 267–273. doi: 10.1038/ng1180 12808457

[B33] Muoio D. M. (2014). Metabolic inflexibility: When mitochondrial indecision leads to metabolic gridlock. Cell. 159, 1253–1262. doi: 10.1016/j.cell.2014.11.034 25480291 PMC4765362

[B34] Nicholls D. G. Ferguson S. J. (2013). Bioenergetics (Boston: Academic Press).

[B35] Picard M. McEwen B. S. (2018). Psychological stress and mitochondria: A conceptual framework. Psychosomatic Med. 80, 126–140. doi: 10.1097/psy.0000000000000544 29389735 PMC5901651

[B36] Picard M. McEwen B. Epel E. Sandi C. (2018). An energetic view of stress: Focus on mitochondria. Front. Neuroendocrinol. 49, 72–85. doi: 10.1016/j.yfrne.2018.01.001 29339091 PMC5964020

[B37] Picard M. Shirihai O. S. (2022). Mitochondrial signal transduction. Cell Metab. 34, 1620–1653. doi: 10.1016/j.cmet.2022.10.008 36323233 PMC9692202

[B38] Picard M. Wallace D. C. Burelle Y. (2016). The rise of mitochondria in medicine. Mitochondrion 30, 105–116. doi: 10.1016/j.mito.2016.07.003 27423788 PMC5023480

[B39] Picca A. Lozanoska-Ochser B. Calvani R. Coelho-Júnior H. J. Leewenburgh C. Marzetti E. (2023). Inflammatory, mitochondrial, and senescence-related markers: Underlying biological pathways of muscle aging and new therapeutic targets. Exp. Gerontology 178, 112204. doi: 10.1016/j.exger.2023.112204 37169101

[B40] Rappaport S. M. (2016). Genetic factors are not the major causes of chronic diseases. PloS One 11, e0154387. doi: 10.1371/journal.pone.0154387 27105432 PMC4841510

[B41] Roca-Portoles A. Tait S. W. G. (2021). Mitochondrial quality control: From molecule to organelle. Cell. Mol. Life Sci. 78, 3853–3866. doi: 10.1007/s00018-021-03775-0 33782711 PMC8106605

[B42] Rohrer B. Bandyopadhyay M. Beeson C. (2016). Reduced metabolic capacity in aged primary retinal pigment epithelium (RPE) is correlated with increased susceptibility to oxidative stress. Adv. Exp. Med. Biol. 854, 793–798. doi: 10.1007/978-3-319-17121-0_106 26427491

[B43] Skalka G. L. Tsakovska M. Murphy D. J. (2024). Kinase signalling adaptation supports dysfunctional mitochondria in disease. Front. Mol. Biosci. 11, 1354682. doi: 10.3389/fmolb.2024.1354682 38434478 PMC10906720

[B44] Spinelli J. B. Haigis M. C. (2018). The multifaceted contributions of mitochondria to cellular metabolism. Nat. Cell Biol. 20, 745–754. doi: 10.1038/s41556-018-0124-1 29950572 PMC6541229

[B45] Sterling P. Eyer J. (1988). “ Allostasis: A new paradigm to explain arousal pathology,” in Handbook of life stress, cognition and health ( John Wiley & Sons, Oxford, England), 629–649.

[B46] Sun N. Youle R. J. Finkel T. (2016). The mitochondrial basis of aging. Mol. Cell 61, 654–666. doi: 10.1016/j.molcel.2016.01.028 26942670 PMC4779179

[B47] Terman A. Brunk U. T. (2004). Myocyte aging and mitochondrial turnover. Exp. Gerontology 39, 701–705. doi: 10.1016/j.exger.2004.01.005 15130664

[B48] Tippairote T. Bjørklund G. Yaovapak A. (2022). The continuum of disrupted metabolic tempo, mitochondrial substrate congestion, and metabolic gridlock toward the development of non-communicable diseases. Crit. Rev. Food Sci. Nutr. 62, 6837–6853. doi: 10.1080/10408398.2021.1907299 33797995

[B49] Tippairote T. Hoonkaew P. Suksawang A. Tippairote P. (2025). From adaptation to exhaustion: Defining exposure-related malnutrition as a bioenergetic phenotype of aging. Biogerontology 26, 161. doi: 10.1007/s10522-025-10302-2 40802114

[B50] Tsui K.-H. Cheng S.-H. Wang B. Lin P.-H. Raj E. N. Lin L.-T. . (2026). Mitochondrial quality control as a central pharmacological target in aging. Pharmacol. Res. 227, 108188. doi: 10.1016/j.phrs.2026.108188 41956139

[B51] Wallace D. C. (2013). A mitochondrial bioenergetic etiology of disease. J. Clin. Invest. 123, 1405–1412. doi: 10.1172/jci61398 23543062 PMC3614529

[B52] Wang X. Zhang G. (2025). The mitochondrial integrated stress response: A novel approach to anti-aging and pro-longevity. Ageing Res. Rev. 103, 102603. doi: 10.1016/j.arr.2024.102603 39608727

[B53] Xu X. Pang Y. Fan X. (2025). Mitochondria in oxidative stress, inflammation and aging: From mechanisms to therapeutic advances. Signal Transduction Targeted Ther. 10, 190. doi: 10.1038/s41392-025-02253-4 40500258 PMC12159213

[B54] Xu K. Saaoud F. Shao Y. Lu Y. Yang Q. Jiang X. . (2024). A new paradigm in intracellular immunology: Mitochondria emerging as leading immune organelles. Redox Biol. 76, 103331. doi: 10.1016/j.redox.2024.103331 39216270 PMC11402145

[B55] Zampino M. Tanaka T. Ubaida-Mohien C. Fantoni G. Candia J. Semba R. D. . (2020). A plasma proteomic signature of skeletal muscle mitochondrial function. Int. J. Mol. Sci. 21, 9540. doi: 10.3390/ijms21249540 33333910 PMC7765442

[B56] Zhang X. Gao Y. Zhang S. Wang Y. Pei X. Chen Y. . (2025). Mitochondrial dysfunction in the regulation of aging and aging-related diseases. Cell Commun. Signaling 23, 290. doi: 10.1186/s12964-025-02308-7 40537801 PMC12177975

