# Supplementary figures and images for: Mapping bioenergetic mechanisms across aging hallmarks: a systematic evidence map and secondary conceptual synthesis
Source: Front Physiol. 2026 Jun 11;17:1868353. doi: 10.3389/fphys.2026.1868353 (PMC13293893; doi:10.3389/fphys.2026.1868353)

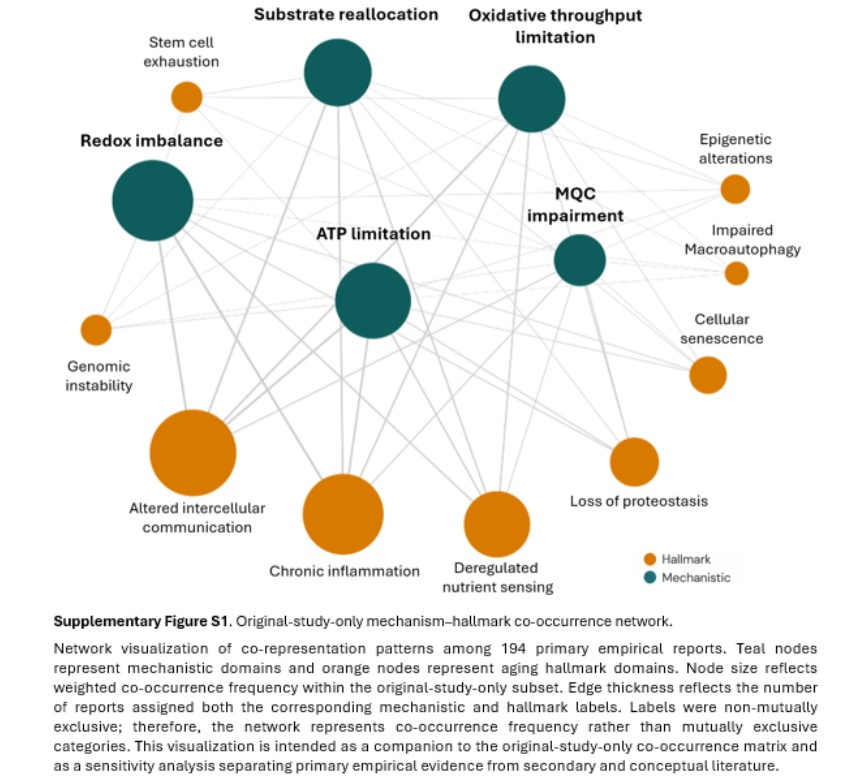

Supplement: Supplementary file 1 [file Image1.tif]
