# Supplementary material for: Mapping bioenergetic mechanisms across aging hallmarks: a systematic evidence map and secondary conceptual synthesis
Source: Front Physiol. 2026 Jun 11;17:1868353. doi: 10.3389/fphys.2026.1868353 (PMC13293893; doi:10.3389/fphys.2026.1868353)
Supplement: Supplementary file 2 [file Table1.docx]

**Supplementary File S1. Completed PRISMA 2020 Checklist**

**Manuscript:** Mapping Bioenergetic Constraints Across Aging Hallmarks: A Systematic Review, Evidence Map, and Secondary Conceptual Synthesis

**Note:** Locations are reported by manuscript section, figure, table, and supplementary file because final page and line numbers are not yet available. Page and line numbers should be updated after final manuscript formatting.

Checklist source: PRISMA 2020 checklist adapted from Page et al. (2021), licensed under CC BY 4.0.

| **Section and topic** | **Item #** | **Checklist item** | **Location where item is reported in the current manuscript** |
| --- | --- | --- | --- |
| TITLE | 1 | Identify the report as a systematic review. | Title page / manuscript title. The title identifies the article as a systematic review. |
| ABSTRACT | 2 | See the PRISMA 2020 for Abstracts checklist. | Abstract. The abstract reports background, methods, results, and conclusion, including structured evidence mapping, use of a previously curated dataset, final synthesis set of 433 reports, main mapped findings, and cautious hypothesis-generating interpretation. |
| INTRODUCTION: Rationale | 3 | Describe the rationale for the review in the context of existing knowledge. | Section 1, Introduction. The rationale is provided by explaining that mitochondrial dysfunction is widely recognized in aging but remains mechanistically heterogeneous, and that the bioenergetic basis linking aging hallmark domains remains incompletely defined. |
| INTRODUCTION: Objectives | 4 | Provide an explicit statement of the objective(s) or question(s) the review addresses. | Section 2, Study Objectives and Research Questions. The primary objective, secondary objectives, and seven research questions are stated explicitly. |
| METHODS: Eligibility criteria | 5 | Specify the inclusion and exclusion criteria for the review and how studies were grouped for the syntheses. | Sections 3.3, Conceptual framework; 3.5, Eligibility Criteria; 3.8, Evidence labeling framework; and 3.10, Data synthesis and evidence mapping. Reports were grouped by aging hallmark domain, stage of mitochondrial or bioenergetic impairment, mechanistic domain, evidence type, and evidence tier. |
| METHODS: Information sources | 6 | Specify all databases, registers, websites, organisations, reference lists and other sources searched or consulted to identify studies. Specify the date when each source was last searched or consulted. | Section 3.4, Information sources and search strategy; Figure 1; Supplementary Table S1. Database searches were conducted in PubMed, Scopus, Web of Science Core Collection, and the Cochrane Library. The final database search was completed on May 15, 2025. Forward and backward citation searching, final update checks, and evidence-map verification were also used as described in Methods. |
| METHODS: Search strategy | 7 | Present the full search strategies for all databases, registers and websites, including any filters and limits used. | Section 3.4, Information sources and search strategy; Supplementary Table S1, full search strategies. Database searches were limited to reports published between 2005 and 2025. Selected foundational or newly available high-yield mechanistic reports outside this window were retained only when identified through citation searching, final update checks, or evidence-map verification. |
| METHODS: Selection process | 8 | Specify the methods used to decide whether a study met the inclusion criteria of the review, including how many reviewers screened each record and each report retrieved, whether they worked independently, and if applicable, details of automation tools used in the process. | Section 3.6, Study Selection and Screening Process. Search results were imported into Rayyan for de-duplication and screening management. Two independent reviewers screened titles/abstracts and full-text reports, with disagreements resolved by discussion and third-reviewer adjudication when needed. Rayyan supported organization and de-duplication but did not replace reviewer eligibility decisions. |
| METHODS: Data collection process | 9 | Specify the methods used to collect data from reports, including how many reviewers collected data from each report, whether they worked independently, any processes for obtaining or confirming data from study investigators, and if applicable, details of automation tools used in the process. | Section 3.7, Data extraction. Data were extracted using a standardized and piloted extraction form by two independent reviewers, with discrepancies resolved by consensus. Missing or unclear information was recorded as not available. |
| METHODS: Data items | 10a | List and define all outcomes for which data were sought. Specify whether all results compatible with each outcome domain in each study were sought, and if not, the methods used to decide which results to collect. | Sections 3.7, Data extraction; 3.8, Evidence labeling framework; and Supplementary Table S3. Because the review is an evidence map rather than an intervention-effect review, predefined data domains were mechanistic and conceptual rather than clinical effect outcomes. |
| METHODS: Data items | 10b | List and define all other variables for which data were sought. Describe any assumptions made about any missing or unclear information. | Section 3.7, Data extraction; Section 3.8, Evidence labeling framework; Supplementary Table S3. Extracted variables included bibliographic information, study characteristics, biological model, mitochondrial and bioenergetic variables, aging hallmark-related processes, mechanistic findings, outcomes where applicable, interventions/exposures, confounders, study limitations, and reviewer notes. Missing or unclear information was recorded as not available. |
| METHODS: Study risk of bias assessment | 11 | Specify the methods used to assess risk of bias in the included studies, including details of the tool(s) used, how many reviewers assessed each study and whether they worked independently, and if applicable, details of automation tools used in the process. | Section 3.12, Risk of bias, reporting bias, and certainty assessment. Formal study-level risk-of-bias assessment using a single standardized tool was not performed because the evidence base included heterogeneous original human, animal, cellular, review-based, and conceptual reports. Evidence tiering was used to contextualize mechanistic contribution and inferential strength. |
| METHODS: Effect measures | 12 | Specify for each outcome the effect measure(s) used in the synthesis or presentation of results. | Section 3.10, Data synthesis and evidence mapping. No pooled effect estimates were calculated. Results are presented as descriptive counts and proportions of non-mutually exclusive labels and as narrative synthesis. |
| METHODS: Synthesis methods | 13a | Describe the processes used to decide which studies were eligible for each synthesis. | Sections 3.5, Eligibility Criteria; 3.8, Evidence labeling framework; and 3.10, Data synthesis and evidence mapping. Included reports were eligible for synthesis when they described mitochondrial or bioenergetic mechanisms relevant to aging hallmark domains and could be classified using the evidence-map framework. |
| METHODS: Synthesis methods | 13b | Describe any methods required to prepare the data for presentation or synthesis, such as handling missing summary statistics or data conversions. | Sections 3.7, Data extraction; 3.8, Evidence labeling framework; and 3.10, Data synthesis and evidence mapping. Non-mutually exclusive labels were assigned to reports, and descriptive counts and proportions were calculated for report-level label associations. |
| METHODS: Synthesis methods | 13c | Describe any methods used to tabulate or visually display results of individual studies and syntheses. | Sections 3.10, Data synthesis and evidence mapping; 3.11, Graphical and network visualization methods; Figures 1-6; Supplementary Table S3. Evidence-map findings were displayed using a PRISMA flow diagram, horizontal bar charts, absolute and proportional stacked bar charts, and a co-occurrence network. |
| METHODS: Synthesis methods | 13d | Describe any methods used to synthesize results and provide a rationale for the choice(s). If meta-analysis was performed, describe the model(s), heterogeneity methods, and software package(s) used. | Sections 3.2, Study Design; 3.10, Data synthesis and evidence mapping; and 3.11, Graphical and network visualization methods. Quantitative meta-analysis was not performed because of substantial heterogeneity in report type, biological model, outcome measures, and mechanistic focus. Structured evidence mapping and narrative synthesis were used instead. |
| METHODS: Synthesis methods | 13e | Describe any methods used to explore possible causes of heterogeneity among study results. | Sections 3.10, Data synthesis and evidence mapping; and 3.13, Sensitivity and heterogeneity assessment. Heterogeneity was explored descriptively by evidence type, biological model, hallmark domain, mechanistic domain, stage of impairment, evidence tier, population, disease context, measurement approach, and level of inference. |
| METHODS: Synthesis methods | 13f | Describe any sensitivity analyses conducted to assess robustness of the synthesized results. | Section 3.13, Sensitivity and heterogeneity assessment. No formal statistical sensitivity analyses were conducted because the synthesis was descriptive and evidence-map based. Robustness was considered qualitatively by comparing distributions across evidence type, biological model, hallmark domain, mechanistic domain, stage of impairment, and evidence tier. |
| METHODS: Reporting bias assessment | 14 | Describe any methods used to assess risk of bias due to missing results in a synthesis. | Section 3.12, Risk of bias, reporting bias, and certainty assessment. Formal reporting-bias or publication-bias assessment was not performed because no quantitative meta-analysis or pooled effect estimate was generated. |
| METHODS: Certainty assessment | 15 | Describe any methods used to assess certainty or confidence in the body of evidence for an outcome. | Sections 3.9, Evidence tiering; and 3.12, Risk of bias, reporting bias, and certainty assessment. GRADE was not applied because the review did not evaluate a single intervention, comparator, or outcome suitable for certainty grading. Confidence was discussed narratively in relation to evidence tier, study type, biological model, consistency of mechanistic patterns, and limitations. |
| RESULTS: Study selection | 16a | Describe the results of the search and selection process, from the number of records identified to the number of studies included, ideally using a flow diagram. | Section 4.1, Study Selection; Figure 1, PRISMA 2020 flow diagram. Database searches identified 646 records and citation searching identified 52 additional reports. The final synthesis included 433 reports: 408 from database searches and 25 from citation searching. |
| RESULTS: Study selection | 16b | Cite studies that might appear to meet the inclusion criteria, but which were excluded, and explain why they were excluded. | Section 3.6, Study Selection and Screening Process; Section 3.14, Data and materials availability; Figure 1; Supplementary Table S2. Exclusion reasons were not systematically recorded for all title/abstract exclusions. Supplementary Table S2 provides selected examples of excluded reports with reasons, focusing on reports that appeared potentially eligible or required closer adjudication, and should not be interpreted as a complete exclusion-reason log for all excluded records. |
| RESULTS: Study characteristics | 17 | Cite each included study and present its characteristics. | Section 4.2, Characteristics of the included evidence base; Supplementary Table S3. Included-report characteristics, evidence type, biological model, evidence tier, and coding variables are provided in Supplementary Table S3. |
| RESULTS: Risk of bias in studies | 18 | Present assessments of risk of bias for each included study. | Section 3.12, Risk of bias, reporting bias, and certainty assessment; Section 5.6, Boundary conditions and future directions. A formal study-level risk-of-bias table was not produced because of evidence-type heterogeneity. Evidence tier and study design were used instead to contextualize inferential strength. |
| RESULTS: Results of individual studies | 19 | For all outcomes, present, for each study: summary statistics for each group where appropriate and an effect estimate and its precision, ideally using structured tables or plots. | Not applicable to pooled outcome synthesis. The review reports evidence-map label frequencies and narrative synthesis rather than study-level effect estimates. Included-report characteristics, label matrix, and co-occurrence matrix are provided in Supplementary Table S3. |
| RESULTS: Results of syntheses | 20a | For each synthesis, briefly summarize the characteristics and risk of bias among contributing studies. | Sections 4.2-4.8. Evidence type and evidence-tier distributions are summarized in Section 4.2 and Figure 2. Hallmark distribution, stage distribution, mechanistic-domain distribution, co-occurrence, evidence gaps, and summary findings are reported in Sections 4.3-4.8 and Figures 3-5. |
| RESULTS: Results of syntheses | 20b | Present results of all statistical syntheses conducted. If meta-analysis was done, present each summary estimate, precision, and heterogeneity; if comparing groups, describe direction of effect. | Not applicable. No meta-analysis or pooled statistical synthesis was performed. Results are presented as descriptive evidence-map frequencies, proportions, and network co-occurrence patterns in Sections 4.2-4.8 and Figures 2-5. |
| RESULTS: Results of syntheses | 20c | Present results of all investigations of possible causes of heterogeneity among study results. | Sections 4.2-4.7 and 5.6. Heterogeneity and distributional imbalances are considered across evidence type, evidence tier, biological model, hallmark domain, mechanistic domain, stage distribution, and underrepresented domains. |
| RESULTS: Results of syntheses | 20d | Present results of all sensitivity analyses conducted to assess robustness of the synthesized results. | Not applicable. No statistical sensitivity analyses were conducted because the review used descriptive evidence mapping rather than pooled quantitative synthesis. Rationale is provided in Section 3.13. |
| RESULTS: Reporting biases | 21 | Present assessments of risk of bias due to missing results for each synthesis assessed. | Not applicable. Formal reporting-bias assessment was not conducted because no outcome-specific quantitative synthesis was performed. Related limitations are addressed in Sections 3.12 and 5.6. |
| RESULTS: Certainty of evidence | 22 | Present assessments of certainty or confidence in the body of evidence for each outcome assessed. | Section 4.2 and Figure 2B report evidence-tier distribution. Section 5.1 and 5.6 interpret confidence cautiously, emphasizing evidence-map convergence rather than causal proof. GRADE certainty ratings were not applied. |
| DISCUSSION | 23a | Provide a general interpretation of the results in the context of other evidence. | Sections 5.1-5.5, Discussion. The principal findings and interpretation are discussed in relation to mitochondrial signaling, nutrient sensing, inflammation, intercellular communication, staged bioenergetic impairment, and cross-domain organization. |
| DISCUSSION | 23b | Discuss any limitations of the evidence included in the review. | Sections 5.1 and 5.6. Limitations of the included evidence include heterogeneity, substantial review-based/contextual literature, limited direct evidence, sparse longitudinal/interventional evidence, uneven hallmark representation, and inability to infer causality or temporal sequence. |
| DISCUSSION | 23c | Discuss any limitations of the review processes used. | Sections 3.4, 3.6, 3.12, 3.15, and 5.6. Limitations include secondary evidence-map synthesis of a previously curated mechanism-oriented dataset, no new hallmark-by-hallmark database search, selected rather than complete exclusion-reason reporting in Supplementary Table S2, no formal study-level risk-of-bias assessment, no meta-analysis, and no formal certainty grading. |
| DISCUSSION | 23d | Discuss implications of the results for practice, policy, and future research. | Sections 5.5, 5.6, and 6, Conclusion. Implications are framed cautiously as hypothesis-generating. Future research priorities include longitudinal human studies, tissue-specific studies, intervention studies testing bioenergetic recovery, and experimental models distinguishing functional limitation, adaptive remodeling, and structural impairment over time. |
| OTHER INFORMATION: Registration and protocol | 24a | Provide registration information for the review, including register name and registration number, or state that the review was not registered. | Section 3.1, Protocol and Registration. The review was registered with PROSPERO under registration number CRD420251033154. |
| OTHER INFORMATION: Registration and protocol | 24b | Indicate where the review protocol can be accessed, or state that a protocol was not prepared. | Section 3.1, Protocol and Registration. The manuscript reports PROSPERO registration. The registered protocol is available through PROSPERO under registration number CRD420251033154.. |
| OTHER INFORMATION: Registration and protocol | 24c | Describe and explain any amendments to information provided at registration or in the protocol. | Section 3.1, Protocol and Registration; Section 3.15, Methodological considerations. The main protocol adaptations were reclassification of the previously curated dataset according to aging hallmark domains, addition of the functional-adaptive-structural staging framework, and use of descriptive co-occurrence mapping rather than quantitative synthesis. Retention of selected foundational or newly available reports outside the 2005-2025 database-search window is also described as a methodological adaptation of the evidence-map approach. |
| OTHER INFORMATION: Support | 25 | Describe sources of financial or non-financial support for the review, and the role of the funders or sponsors in the review. | Funding statement. The manuscript states that no specific financial support was received for this review. |
| OTHER INFORMATION: Competing interests | 26 | Declare any competing interests of review authors. | Competing interests statement. The manuscript states that the authors declare no competing interests. |
| OTHER INFORMATION: Availability of data, code and other materials | 27 | Report which materials are publicly available and where they can be found: data collection forms, extracted data, analytic code, and other materials used in the review. | Section 3.14, Data and materials availability; Supplementary materials statement. Supplementary Table S1 provides full database search strategies. Supplementary Table S2 provides selected excluded reports with reasons. Supplementary Table S3 provides the data extraction template, evidence-labeling framework, evidence-tiering framework, included-report characteristics, label matrix, co-occurrence matrix, and figure-generation notes. Additional source-level files or coding logs are available from the corresponding author upon reasonable request, subject to database export and copyright restrictions. |

Abbreviations: ATP, adenosine triphosphate; GRADE, Grading of Recommendations Assessment, Development and Evaluation; PRISMA, Preferred Reporting Items for Systematic Reviews and Meta-Analyses; PROSPERO, International Prospective Register of Systematic Reviews.
